# Extracellular vesicle-based therapeutic strategies for spinal cord injury

**DOI:** 10.20517/evcna.2025.142

**Published:** 2026-02-26

**Authors:** Jingsong Liu, Xuqiang Gong, Yuanliang Sun, Yangyang Wang, Yansong Wang

**Affiliations:** ^1^Department of Orthopedic Surgery, The First Affiliated Hospital of Harbin Medical University, Harbin Medical University, Harbin 150000, Heilongjiang, China.; ^2^The Key Laboratory of Myocardial Ischemia, Ministry of Education, Harbin Medical University, Harbin 150000, Heilongjiang, China.; ^3^NHC Key Laboratory of Cell Transplantation, Harbin Medical University, Harbin 150000, Heilongjiang, China.; ^#^These authors contributed equally to this work.

**Keywords:** Spinal cord injury, exosomes, nerve repair, immune microenvironment

## Abstract

Spinal cord injury (SCI) is a highly disabling disorder of the central nervous system for which no curative therapy is currently available. In recent years, extracellular vesicles - particularly exosomes - have been investigated as cell-free therapeutic approaches in experimental models, owing to their low immunogenicity, favorable biocompatibility and capacity to traverse the blood-spinal cord barrier under specific conditions or delivery routes. This Review summarizes the therapeutic activities and mechanisms of exosomes from diverse sources - including mesenchymal stem cells, immune cells and neural cells - in SCI repair. Reported mechanisms include modulation of the inflammatory microenvironment; inhibition of apoptosis and pyroptosis; mitigation of ferroptosis; promotion of angiogenesis and axonal regeneration; and restriction of glial scar formation. We also discuss advances aimed at enhancing exosome efficacy through cell preconditioning, engineering strategies and integration with biomaterials. Although exosome-based approaches are promising, challenges remain in standardization, targeted delivery and long-term safety. Future work should elucidate the underlying mechanisms and advance clinical translation to robustly evaluate the therapeutic potential of exosomes for SCI repair.

## INTRODUCTION

Spinal cord injury (SCI) is a severe disorder of the central nervous system for which no curative therapy is available. It substantially impairs patients’ quality of life and places major burdens on health systems and economies. Recent estimates indicate that China has 759,302 people living with traumatic SCI, with 66,374 new cases annually^[1]^. In the United States (US), 17,000 new cases occur each year; first-year costs for high-level tetraplegia can exceed US$ 1 million^[2]^. Many patients experience persistent motor, sensory and autonomic dysfunction after injury. Although early interventions - including surgical decompression, hemodynamic stabilization and high-dose corticosteroids - reduce mortality, their impact on functional recovery remains limited^[3,4]^. Restoring neural function remains a central challenge for neuroscience and regenerative medicine.

**Figure 1:**
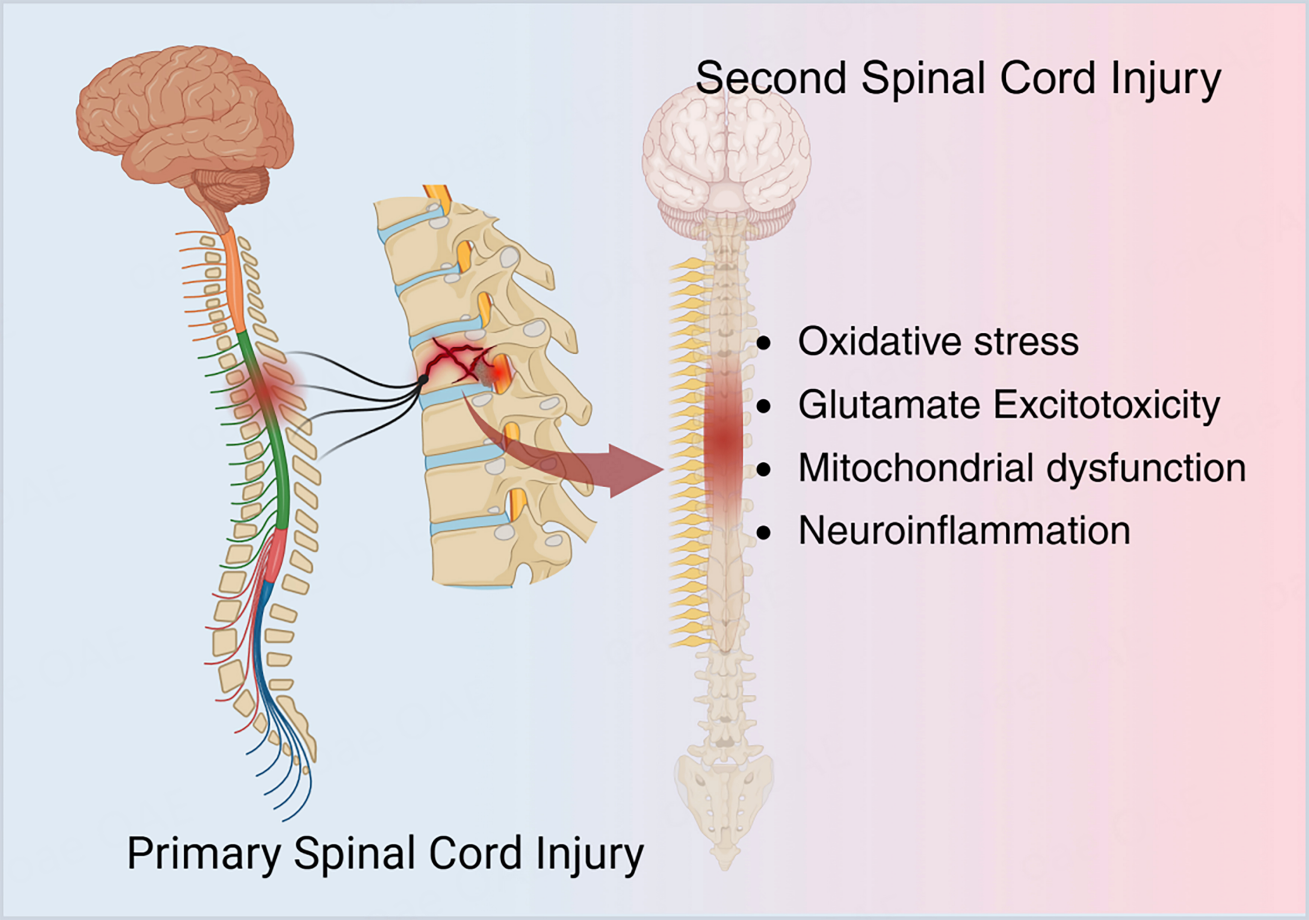
Pathological mechanism of spinal cord injury [Created in BioRender. Liu, J. (2026). https://BioRender.com/bwj2qsb].

SCI remains a major therapeutic challenge owing to a complex spatiotemporal pathological cascade^[5]^. This cascade is often delineated into acute and chronic phases^[5]^. The acute phase is initiated by the primary mechanical insult, which disrupts spinal cord architecture and causes edema, impaired perfusion, ischemia and hypoxia, thereby precipitating a cellular energy crisis^[6]^. Secondary injury mechanisms then propagate tissue damage, including oxidative stress, glutamate excitotoxicity, mitochondrial dysfunction, neuroinflammation and breakdown of blood-spinal cord barrier (BSCB)^[6]^. These processes may persist for weeks to months and culminate in neuronal apoptosis, axonal degeneration, demyelination, circuit disruption and glial scar formation^[5]^. Together, these changes establish a microenvironment unfavorable to regeneration [Figure 1]. Given the limited regenerative capacity of adult central nervous system neurons, the reestablishment of functional synaptic connections by axons is often constrained, which limits neurological recovery^[4,7]^.

Cell-based approaches are under active investigation as potential treatments for SCI. Various cell types - including mesenchymal stem cells (MSCs), neural stem/progenitor cells (NSCs/NPCs), Schwann cells (SCs), and olfactory ensheathing cells - have been evaluated in preclinical and early clinical studies^[8]^. However, evidence indicates that although cell transplantation shows therapeutic potential, it faces several challenges, including limited post-transplant survival and engraftment, immune rejection, risk of tumorigenicity, and ethical considerations^[9]^. Increasing evidence indicates that the therapeutic effects of stem cells are mediated, to a substantial extent, by paracrine mechanisms - including extracellular vesicles (EVs) such as exosomes - rather than by direct neuronal replacement^[9]^.

EVs are membrane-bound particles actively released by cells, with an aqueous lumen that carries a diverse array of bioactive molecules derived from their parent cells, such as proteins, lipids, nucleic acids [including messenger RNAs (mRNAs), microRNAs (miRNAs), long noncoding RNAs (lncRNAs), and circular RNAs (circRNAs)], and metabolites. This molecular cargo highlights their diversity^[10]^. EVs are detected in multiple biofluids, including blood, urine, saliva, cerebrospinal fluid and breast milk. They carry cell- and tissue-associated molecular signatures and act as mediators of intercellular communication. Through these roles, EVs participate in and regulate a broad spectrum of physiological and pathological processes - such as immune responses, tissue regeneration, tumor metastasis and neurodegenerative diseases - supporting their diagnostic and therapeutic potential^[11]^. Based on their biogenesis and release mechanisms, EVs are commonly categorized into several subtypes, including exosomes, microvesicles (MVs; also termed ectosomes) and apoptotic bodies. Among these, exosomes - the most widely studied subtype - are typically 30-150 nm in diameter^[12]^. They originate from the inward budding of the endosomal membrane, forming early endosomes that mature into multivesicular bodies (MVBs) via Endosomal Sorting Complex Required for Transport (ESCRT)-dependent or ESCRT-independent mechanisms. Exosomes are released into the extracellular space when MVBs fuse with the plasma membrane, resulting in the secretion of intraluminal vesicles (ILVs). The surface of exosomes is enriched with commonly used marker molecules - such as tetraspanins (CD9, CD63, CD81), heat-shock proteins (HSP70, HSP90), Tumor Susceptibility Gene 101 (TSG101) and Alix - that are widely used for enrichment and characterization^[12]^.

Owing to their generally low immunogenicity and favorable biocompatibility, ability to cross the blood-brain and BSCB, and potential as acellular therapeutics, EVs - particularly exosomes - are being actively investigated as biotherapeutic vectors^[13]^. They show potential for applications in the research and treatment of central nervous system disorders, including SCI.

Several recent reviews have summarized the emerging roles of EVs in SCI, often organizing the literature by cell source or therapeutic outcome. Building on these efforts, the present review places particular emphasis on how exosomes derived from different cellular origins influence shared biological processes through distinct, context-dependent mechanisms. Rather than treating individual cargos or signaling pathways in isolation, we discuss how variations in cargo composition, recipient cell responsiveness, and injury microenvironment shape the downstream effects of exosome-mediated communication.

In addition, this review highlights recent advances in exosome engineering, preconditioning strategies, and biomaterial-assisted delivery, while critically addressing translational challenges such as experimental heterogeneity, safety considerations, and clinical feasibility. By combining source-specific evidence with a focused discussion of mechanistic patterns and limitations, this work aims to provide readers with a coherent view of current progress and remaining challenges in the development of exosome-based therapies for SCI.

## THERAPEUTIC EFFECTS AND MECHANISMS OF MESENCHYMAL STEM CELL-DERIVED EXOSOMES IN SCI

The therapeutic effects of MSCs have been ascribed, in part, to their secreted exosomes (MSC-Exos). MSC-Exos are nanoscale EVs that carry bioactive molecules such as proteins and nucleic acids, recapitulating key paracrine activities of MSCs while offering practical advantages, including noninvasive administration, amenability to storage, and a lack of replicative capacity that reduces concerns about tumorigenicity^[9,14,15]^. Following SCI, MSC-Exos have been reported to modulate multiple key processes during the secondary injury phase, including microglial and macrophage polarization, attenuation of neuronal and oligodendroglial apoptosis, preservation of BSCB integrity, promotion of axonal regeneration and neurogenesis, and modulation of astroglial scarring, thereby supporting neurological recovery (representative examples are summarized in Table 1, and a more comprehensive overview is provided in Supplementary Table 1).

**Table 1:** Representative therapeutic effects of exosomes derived from different mesenchymal stem cell sources in spinal cord injury

**Exo sources**	**Effects of Exo-administration**	**Animals**	**Ref.**
BM-MSC	miR-24-3p targets MAPK9 to inhibit the JNK/c-Jun/c-Fos pathway; suppresses apoptosis and inflammation	Rat	[16]
UC-MSC	miR-340-5p inhibits the JAK/STAT3 pathway; promotes microglial polarization from M1 to M2	Mouse	[17]
PMSC	miR-125a-3p reduces neutrophil extracellular trap formation and neutrophil activation; attenuates inflammation	Mouse	[18]
ADSC	lncRNA RMRP modulates the EIF4A3/SIRT1 axis; reduces microglial pyroptosis	Mouse	[19]

BM-MSC: Bone marrow mesenchymal stem cell; UC-MSC: umbilical cord mesenchymal stem cell; PMSC: placenta-derived mesenchymal stem cell; ADSC: adipose-derived mesenchymal stem cell; MAPK9: mitogen-activated protein kinase 9; JNK: c-Jun N-terminal kinase; JAK: Janus kinase; STAT3: signal transducer and activator of transcription 3; lncRNA: long non-coding RNA; RMRP: RNA component of mitochondrial RNA processing endoribonuclease; EIF4A3: eukaryotic translation initiation factor 4A3; SIRT1: sirtuin 1.

## INFLAMMATION-MODULATING ROLES AND MECHANISMS OF MSC-EXOS

Following SCI, chemokines and damage-associated molecular patterns (DAMPs) rapidly recruit microglia and monocyte-derived macrophages to the lesion core, biasing their polarization toward an M1-like proinflammatory phenotype. These activated cells upregulate and release proinflammatory cytokines [e.g., tumor necrosis factor α (TNF-α), interleukin (IL)-1β] and express inducible nitric oxide synthase (iNOS), thereby exacerbating secondary injury^[20]^. Research indicates that MSC-Exos modulate the M1-M2 polarization balance by tuning multiple signaling pathways, including Toll-like receptor 4 (TLR4)/nuclear factor kappa-light-chain-enhancer of activated B cells (NF-κB), Janus kinase (JAK)/signal transducer and activator of transcription 3 (STAT3) and Mitogen-activated protein kinase (MAPK), thereby alleviating neuroinflammation^[16,17,21,22]^. For instance, umbilical cord-derived MSC-Exos transfer miR-340-5p to suppress the JAK/STAT3 pathway, promoting a shift in microglial polarization from M1 to M2 and reducing local inflammation^[17]^. Similarly, miRNAs enriched in MSC-Exos, such as miR-24-3p and miR-146b, target inflammatory signaling nodes including mitogen-activated protein kinase 9 (MAPK9), also known as c-Jun N-terminal kinase 2 (JNK2), and TLR4/NF-κB, attenuating inflammatory signaling and stabilizing the lesion microenvironment^[16,21]^. Furthermore, MSC-Exos deliver zinc finger and BTB domain-containing protein 4 (ZBTB4) to inhibit Inter-alpha-trypsin inhibitor heavy chain 3 (ITIH3), thereby mitigating astrocyte-induced neuronal damage^[23]^. Another study reported that MSC-Exos reduce the formation of neutrophil extracellular traps (NETs), limiting excessive neutrophil activation and thereby suppressing early secondary inflammation^[18]^. Collectively, these immunophenotypic reprogramming mechanisms are thought to contribute substantially to the neuroprotective effects of MSC-Exos.

## NEUROPROTECTIVE ROLES AND MECHANISMS OF MSC-EXOS

SCI triggers widespread regulated neuronal death - via apoptosis, pyroptosis and ferroptosis - driven by excitotoxicity, inflammatory mediators and disrupted iron homeostasis. Given the limited regenerative capacity of adult central nervous system neurons and the essential role of oligodendrocytes in myelination, protecting surviving cells and slowing the progression of cell death is a priority for sustaining long-term neural transmission after SCI.

Evidence indicates that MSC-Exos deliver functional miRNAs - such as miR-219-5p, miR-497-5p and miR-24-3p - to damaged neurons and glial cells. These miRNAs target key molecules in apoptosis and pyroptosis pathways, including Pellino E3 ubiquitin protein ligase 1 (PELI1), MAPK9 and thioredoxin-interacting protein (TXNIP), thereby inhibiting c-Jun N-terminal kinase (JNK)-c-Jun-c-Fos signaling and NOD-like receptor family pyrin domain containing 3 (NLRP3) inflammasome activation, reducing neuronal loss and myelin damage^[16,24-26]^. Beyond dampening cell-death signaling, MSC-Exos enhance endogenous pro-survival mechanisms - for example, by increasing the B-cell lymphoma 2 (BCL-2)/BCL-2-associated X protein (BAX) ratio and activating the Wnt/β-catenin pathway - thereby improving cellular resilience to injury^[27]^. Ferroptosis - a regulated cell-death modality implicated in secondary injury - is characterized by accumulation of lipid peroxidation and disruption of glutathione metabolism^[28]^. MSC-Exos counteract this process by activating the nuclear factor erythroid 2-related factor 2 (NRF2)-GTP cyclohydrolase 1 (GCH1)-tetrahydrobiopterin (BH4) antioxidant signaling axis, enhancing glutathione synthesis and restoring iron homeostasis, thereby suppressing Lipopolysaccharide (LPS)-induced ferroptotic cascades and preserving neuronal mitochondrial integrity^[25]^. Further work indicates that miR-219-5p within MSC-Exos mitigates glutathione peroxidase 4 (GPX4) downregulation and reactive oxygen species (ROS) bursts via the ubiquitin-conjugating enzyme E2 Z (UBE2Z)/NRF2 axis, demonstrating robust neuroprotective effects in relevant models^[25]^.

Collectively, MSC-Exos act through multiple mechanisms - attenuating apoptosis and pyroptosis, reinforcing pro-survival signaling and limiting ferroptosis - to establish an integrated cytoprotective response that supports recovery after secondary SCI.

## BSCB-PRESERVING ROLES AND MECHANISMS OF MSC-EXOS

The integrity of BSCB is crucial for maintaining spinal cord microenvironmental homeostasis. However, after SCI, sustained exposure to inflammatory mediators, matrix metalloproteinases (MMPs) and ROS contributes to injury of local microvascular endothelial cells and downregulation of tight-junction protein expression. This increases BSCB permeability, promoting vasogenic edema and excessive immune-cell infiltration, thereby exacerbating neural tissue damage^[29]^.

MSC-Exos have been reported to inhibit endothelial apoptosis, promote angiogenesis, support tight-junction reassembly and stabilize the microvascular network. Studies indicate that MSC-Exos enhance transforming growth factor β (TGF-β) expression in M2 macrophages, establishing a bidirectional “immune regulation-BSCB stabilization” axis: this crosstalk mitigates inflammation and, via TGF-β, in turn regulates endothelial junctional complexes to facilitate barrier reconstruction^[30]^. Specifically, MSC-Exos upregulate TGF-β/transforming growth factor-β receptor (TGF-βR) signaling and increase expression of tight-junction proteins such as vascular endothelial (VE)-cadherin, Zonula occludens-1 (ZO-1) and claudin-5, thereby reducing BSCB permeability and limiting infiltration of circulating inflammatory cells^[30]^. Furthermore, by suppressing MMP-2/MMP-9 and endothelin-1 (ET-1) expression, MSC-Exos limit junctional degradation while concurrently enhancing angiogenic signaling [e.g., vascular endothelial growth factor (VEGF)/vascular endothelial growth factor receptor 2 (VEGFR2)]. These actions help establish a lower-permeability microvascular milieu in early SCI, reducing edema and secondary neural injury^[31]^. Additionally, MSC-Exos promote pericyte survival and inhibit pericyte pyroptosis by modulating nucleotide-binding oligomerization domain-containing protein 1 (NOD1) signaling, thereby supporting BSCB integrity^[32]^.

In summary, MSC-Exos mediate BSCB repair through coordinated, multi-pathway actions. Future work should move from systems-level descriptions toward delineating the temporal dynamics and cell-type-specific actions of MSC-Exo cargo, to address key challenges for precise clinical translation.

## AXONAL-REGENERATION AND NEURAL-REPAIR ROLES AND MECHANISMS OF MSC-EXOS

Following SCI, neural circuitry is disrupted by axonal transection and growth-cone collapse. Even with inflammation controlled and cell death attenuated, intrinsic growth programs are often insufficient for axons to overcome the inhibitory microenvironment and re-establish functional connections. Accordingly, enhancing axonal regenerative capacity and modulating endogenous neurogenic potential are priorities through which MSC-Exos may support functional recovery.

Studies indicate that MSC-Exos downregulate axonal growth inhibitors via the miR-431-3p/repulsive guidance molecule A (RGMA) axis, which may relieve inhibitory cues after injury and permit growth-cone reformation and axonal extension^[33]^. Notably, certain MSC-Exo subpopulations (e.g., CD271^+^CD56^+^) deliver neurotrophic cues and upregulate proteins associated with axonal growth and microtubule stability - such as growth-associated protein 43 (GAP43), neurofilament-H (NF200) and microtubule-associated protein 2 (MAP2) - supporting directed axonal outgrowth towards distal targets^[33]^. Beyond effects on mature neurons, MSC-Exos transfer miRNAs such as let-7a-5p, which target the HMGA2/SMAD2 axis to promote differentiation of endogenous NSCs into neurons^[34]^. MSC-Exos also enhance neural stem-cell proliferation via MEK/ERK/CREB signaling and promote endogenous progenitor neurogenesis, thereby contributing to functional recovery^[35]^.

In summary, MSC-Exos act through multimodal, pro-regenerative mechanisms - relieving inhibitory signaling, reinforcing intrinsic axonal growth capacity and activating endogenous neurogenesis - suggesting potential strategies for rebuilding neural circuits after SCI. Future work should prioritize identification of MSC-Exo subpopulations with greater regenerative activity, definition of their key cargo, and development of biomaterial-integrated targeted delivery systems to help advance from structural regrowth to functional recovery.

## GLIAL-SCAR-LIMITING AND MICROENVIRONMENT-REMODELING ROLES AND MECHANISMS OF MSC-EXOS

After SCI, reactive astrocytes - a resident glial population of the central nervous system - become activated and contribute to a scar barrier with enrichment of A1-like astrocytic states^[36]^. This glial scar secretes inhibitory extracellular matrix (ECM) components, such as chondroitin sulfate proteoglycans (CSPGs), which obstruct axonal growth into the lesion core, thereby impeding long-term functional recovery after SCI^[37]^. Accordingly, priorities for improving the regenerative milieu include limiting A1-like activation while promoting A2-like, reparative astrocyte states.

Evidence indicates that MSC-Exos suppress nuclear translocation of NF-κB p65, thereby reducing A1-associated inflammatory signaling and limiting astrocyte-derived deposition of inhibitory ECM components^[38]^. In addition, MSC-Exos modulate astrocytic pyroptosis and inflammatory activation states, attenuating sustained scar formation^[38,39]^. These effects favor a more permissive ‘soft scar’ phenotype with neurotrophic and pro-angiogenic properties, supporting axonal penetration of the lesion border and extension towards distal targets.

By regulating astrocyte phenotypic states, limiting neurotoxic activation and fostering a reparative microenvironment, MSC-Exos provide a multi-target approach to address the inhibitory effects of glial scarring after SCI. Future work should prioritize identification of MSC-Exos subpopulations with greater scar-remodeling activity, definition of key effector molecules and their temporal dynamics, and exploration of synergy with combined therapies - such as biomaterial scaffolds and rehabilitative training - to help advance from structural regrowth to functional recovery.

## COMPARATIVE MECHANISTIC LANDSCAPE OF EXOSOMAL CARGO ACROSS CELL SOURCES

Exosomes derived from different cellular sources have been shown to exert therapeutic effects in SCI through a range of biological processes that are discussed throughout this review. Although these mechanisms are often reported in a source-specific context, several recurring biological themes become apparent when the literature is considered collectively. Across diverse experimental models, exosomes consistently influence post-injury inflammation, regulate multiple forms of programmed cell death, contribute to the maintenance of BSCB integrity, and support axonal growth and tissue repair.

At the molecular level, these effects are mediated by heterogeneous cargos, including miRNAs, lncRNAs, proteins, and lipids. Rather than acting through isolated or linear pathways, exosomal cargos typically engage signaling networks whose functional outcomes depend on both their cellular origin and the state of recipient cells within the injured spinal cord. Consequently, cargos associated with similar therapeutic effects may operate through distinct molecular routes. For example, several miRNAs have been reported to attenuate neuroinflammation following SCI, yet their downstream targets and signaling contexts vary according to the parent cell type and the local injury microenvironment.

Importantly, overlap among reported exosomal cargos should not be interpreted simply as functional redundancy. Instead, multiple cargos may modulate related biological processes through partially distinct mechanisms, providing robustness across different stages of injury while allowing source-dependent variation in signaling strength and specificity. Differences in cargo composition, co-packaging, and recipient-cell responsiveness are therefore likely to shape the relative contribution of individual molecules in a given experimental setting.

Taken together, the mechanistic diversity highlighted in this review reflects the context-dependent nature of exosome-mediated regulation in SCI rather than a lack of specificity. Recognizing both shared biological processes and source-related differences among exosomal cargos may help reconcile seemingly divergent findings across studies and inform more rational approaches to the design of exosome-based therapeutic strategies.

## THERAPEUTIC EFFECTS AND MECHANISMS OF IMMUNE CELL-DERIVED EXOSOMES ON SCI

As work on stem cell-derived exosomes continues to illuminate their immunomodulatory and neurorestorative activities, exosomes released by immune cells have attracted growing attention in SCI research. As central regulators of inflammation and tissue repair, immune cell-derived exosomes participate in early post-injury immune cascades and, through signaling and miRNA transfer, contribute - in experimental models - to neuroprotection and functional recovery during later repair phases. Current studies focus largely on exosomes from macrophages, T cells and microglia, which exhibit distinct yet complementary roles in inflammation control, angiogenesis and neuronal support. The following section summarizes key advances and mechanistic insights into immune cell-derived exosomes in SCI repair (representative examples are summarized in Table 2, and a more comprehensive overview is provided in Supplementary Table 2).

**Table 2:** Representative therapeutic effects of Exos isolated from different immune cells in spinal cord injury

**Exo sources**	**Effects of Exo-administration**	**Animals**	**Ref.**
Macrophage	Suppresses AIM2/ASC/Caspase-1 inflammasome; reduces neuronal pyroptosis; promotes axonal regeneration and motor recovery	Mouse	[40]
M1-Macrophage	miR-155 activates NF-κB signaling, inducing EndoMT and mitochondrial dysfunction in endothelial cells after SCI	Mouse	[41]
M2-Macrophage	Activates HIF-1α/VEGF axis to enhance post-injury angiogenesis and functional recovery	Rat	[42]
Regulatory T cells	Inhibit PI3K/Akt/mTOR pathway to drive anti-inflammatory microglial polarization	Rat	[43]
M2-Microglial	miR-23a-3p/PTEN/PI3K/AKT axis regulates macrophage polarization toward M2 phenotype	Mouse	[44]

Exos: Exosomes; AIM2: absent in melanoma 2; ASC: apoptosis-associated speck-like protein containing a CARD; NF-κB: nuclear factor kappa B; EndoMT: endothelial-to-mesenchymal transition; SCI: spinal cord injury; HIF-1α: hypoxia-inducible factor 1 alpha; VEGF: vascular endothelial growth factor; PI3K: phosphoinositide 3-kinase; Akt: protein kinase B; mTOR: mechanistic target of rapamycin; miR: microRNA; PTEN: phosphatase and tensin homolog.

## MACROPHAGE-DERIVED EXOSOMES (MACROPHAGE-EXOS)

Following SCI, peripheral monocytes and monocyte-derived macrophages are recruited to the lesion site in response to inflammatory chemokines. There, they interact with resident microglia to shape the inflammatory microenvironment. Macrophages are often operationally categorized into M1-like (proinflammatory) and M2-like (reparative) states. Evidence indicates that exosomes from M1-like macrophages activate pathways such as NF-κB, amplifying proinflammatory feedback that can impede tissue repair^[45]^. Conversely, M2 macrophage-derived exosomes are enriched in miRNAs associated with anti-inflammatory and reparative processes. These vesicles have been reported to attenuate upstream proinflammatory signaling (e.g., NF-κB), reduce mediators such as IL-1β and TNF-α, and dampen chemotactic cascades, thereby supporting the transition from the injury-amplification phase to repair^[46]^. In SCI models, exosomes from M2-like macrophages have been reported to enhance angiogenesis and functional recovery via the hypoxia-inducible factor 1 α (HIF-1α)/VEGF axis^[42]^. Furthermore, M2-derived exosomes (M2-Exos) activate Wnt/β-catenin signaling via transfer of the deubiquitinase OTULIN, which promotes post-SCI vascular regeneration and neurological recovery^[47]^. Accordingly, M2 macrophage-derived exosomes are being investigated as potential therapeutic candidates for SCI.

## T CELL-DERIVED EXOSOMES (T CELL-EXOS)

T cells play an important role in maintaining immune balance throughout the subacute to chronic phases of SCI^[48]^. Evidence indicates that exosomes derived from regulatory T cells (Tregs) can deliver miR-709, which targets NF-κB activating protein (NKAP), thereby reducing microglial pyroptosis, tempering the inflammatory response and limiting secondary injury after SCI^[49]^. In addition, Treg-derived exosomes (Treg-Exos) can transfer miR-2861, which suppresses interleukin-1 receptor-associated kinase 1 (IRAK1) expression, thereby enhancing BSCB integrity and improving motor function^[50]^. Recent work suggests that Treg cells modulate microglial cholesterol metabolism via the CTLA-4/Abcg1 pathway, alleviating lipid accumulation and exerting neuroprotective effects that collectively support functional recovery after SCI^[51]^. Consistent with this, we previously reported that modulation of microglial cholesterol metabolism improves motor recovery following SCI^[52]^. Therefore, Treg-Exos that modulate post-SCI lipid metabolism are being investigated as potential therapeutic candidates for SCI.

## MICROGLIAL CELL-DERIVED EXOSOMES (MICROGLIAL-EXOS)

In the early phase of SCI, microglia are rapidly activated and release exosomes that mediate intercellular communication. As the resident macrophage-like cells of the central nervous system, microglia can adopt proinflammatory and anti-inflammatory activation states. Exosomes derived from M2-like (anti-inflammatory/reparative) microglia can limit astrocytic transitions towards A1-like neurotoxic states, reduce the release of proinflammatory mediators, and have been associated with improvements in the lesion microenvironment and behavioral outcomes in animal models^[53]^.

At the molecular level, microglial exosomes are enriched for functional miRNAs. For instance, exosomes from M2-like microglia enriched in miR-151-3p can attenuate neuronal apoptosis and enhance functional recovery after SCI by modulating the tumor protein p53 (p53)/Cyclin-dependent kinase inhibitor 1A (p21)/Cyclin-dependent kinase 1 (CDK1) signaling pathway^[54]^. Further studies suggest that exosomal miR-672-5p promotes repair after traumatic SCI by limiting neuronal pyroptosis mediated by the Absent in melanoma 2 (AIM2)-Apoptosis-associated speck-like protein containing a CARD (ASC)-caspase-1 signaling axis^[40]^.

In summary, immune cell-derived exosomes help to establish a dynamic balance between inflammation resolution, neuroprotection and tissue regeneration by transferring networks of functional miRNAs. These vesicles are being investigated as potential strategies to achieve more precise immune modulation and to support functional recovery following SCI.

## THERAPEUTIC ROLES AND MECHANISMS OF NEURAL CELL-DERIVED EXOSOMES IN SCI

Neural cell-derived exosomes - including those from microglia, neurons, astrocytes, oligodendrocytes, SCs and olfactory ensheathing cells - are being investigated as therapeutic candidates for SCI. Acting at the level of neuronal and glial circuits, these vesicles carry specific miRNAs, transcription factors and signaling proteins, and promote tissue repair by modulating recipient-cell metabolism and gene expression. For clarity, we refer to the principal subtypes as NSC-derived exosomes (NSC-Exos), neuron-derived exosomes (Neuron-Exos), astrocyte-derived exosomes (Astrocyte-Exos), microglia-derived exosomes (Microglia-Exos), Schwann cell-derived exosomes (SC-Exos), olfactory ensheathing cell-derived exosomes (OEC-Exos) and oligodendrocyte-derived exosomes (Oligo-Exos). The following sections summarize their research progress and mechanisms in SCI repair (representative examples are summarized in Table 3, while a more comprehensive overview is provided in Supplementary Table 3).

**Table 3:** Representative therapeutic effects of Exos isolated from different neural cells in spinal cord injury

**Exo sources**	**Effects of Exo-administration**	**Animals**	**Ref.**
NSC	Differential mRNA/lncRNA/circRNA/miRNA upregulate TSC2 in recipient cells	Mouse	[55]
Neuron	miR-21 activates TGF-β/SMAD2, drives astroglial scarring and demyelination	Rat	[56]
Astrocyte	Reduces pericyte proliferation and fibrotic scar formation	Rat	[57]
SC	Integrin-β1 mediates angiogenesis, neuroprotection, and tissue repair	Rat	[58]
OEC	NF-κB/c-Jun inhibition drives M2 polarization and immune balance	Rat	[59]
Oligodendrocyte	Nogo-A/Nogo-66-NgR1 interaction inhibits axonal regeneration	Mouse	[60]

NSC: Neural stem cell; SC: Schwann cell; OEC: olfactory ensheathing cell; Exo: exosome; mRNA: messenger RNA; lncRNA: long non-coding RNA; circRNA: circular RNA; miRNA: microRNA; TSC2: tuberous sclerosis complex 2; TGF-β: transforming growth factor beta; SMAD2: SMAD family member 2; Integrin-β1: integrin beta 1; NF-κB: nuclear factor kappa B; Nogo-A: neurite outgrowth inhibitor A; Nogo-66: 66-amino-acid extracellular domain of Nogo-A; NgR1: Nogo receptor 1.

## NEURAL STEM CELL-DERIVED EXOSOMES

Neural stem cell-derived exosomes (NSC-EXOS) are among the more widely studied subtypes of neural cell-derived exosomes. Evidence indicates that NSC-Exos modulate neuroprotection, BSCB repair and angiogenesis by tuning signaling pathways such as Phosphoinositide 3-kinase (PI3K)-protein kinase B (AKT) signaling pathway, phosphatase and tensin homolog (PTEN) and nuclear factor erythroid 2-related factor 2 (NRF2)-heme oxygenase-1 (HO-1) signaling pathway. NSC-Exos have been reported to support neuronal survival and attenuate secondary inflammatory responses by inhibiting PTEN and activating the AKT pathway^[61]^. In addition, exosomes derived from necroptotic NSCs after SCI carry differentially expressed mRNAs, lncRNAs, circRNAs and miRNAs, and have been associated with upregulation of Tuberous sclerosis complex 2 (TSC2) in recipient cells and improved functional recovery in experimental models^[55]^.

Overall, NSC-Exos exert neuroprotective effects by regulating inflammation, oxidative stress and cell-survival pathways. Both intravenous and intrathecal administration have been used in preclinical SCI models, with beneficial effects reported and generally acceptable tolerability.

## NEURON-EXOS

Neuron-Exos, directly derived from mature neurons, are important regulators of neural circuit remodeling. Evidence indicates that Neuron-Exos can modulate microglial and astrocytic activation states via specific miRNAs, thereby alleviating inflammation and supporting remyelination. A study reported that miR-124-3p-enriched Neuron-Exos inhibit M1-like microglial and A1-like astrocytic activation, promote a shift towards M2-like states, and reduce inflammation^[62]^. Additionally, miR-21 in Neuron-Exos supports remyelination and neurological recovery by downregulating SMAD family member 7 (SMAD7) and activating the TGF-β/SMAD2 signaling pathway^[56]^.

These findings suggest that Neuron-Exos concurrently modulate inflammatory responses and remyelination through molecular axes such as miR-124-3p/Myosin heavy chain 9 (MYH9) and miR-21/SMAD7, and can also improve mitochondrial function and cellular bioenergetics. Consequently, Neuron-Exos appear to act primarily through miRNA-mediated multitarget regulation, simultaneously suppressing neuroinflammation and promoting remyelination.

## ASTROCYTE-EXOS

Astrocyte-Exos have context-dependent effects in SCI repair. Under homeostatic conditions, astrocyte-Exos can promote regeneration via neurotrophic effects, whereas in reactive/inflammatory states they may amplify inflammatory responses. A study reported that miR-5121-enriched astrocyte-Exos activate the AKT serine/threonine kinase 2 (AKT2)-mechanistic target of rapamycin (mTOR)-ribosomal protein S6 kinase beta-1 (p70S6K) signaling pathway, promoting neurite outgrowth and synapse formation^[57]^. Related work suggested that activation of Hippo signaling - with consequent restraint of Yes-associated protein (YAP) overactivation - improves axonal regeneration^[63]^. However, under inflammatory stimulation, astrocyte-Exos acting via the C-C motif chemokine ligand 2 (CCL2)/C-C motif chemokine receptor 2 (CCR2) signaling axis have been reported to enhance microglial activation and the secretion of IL-1β and TNF-α, exacerbating neuronal damage^[64]^. Furthermore, Rab27a-dependent increases in CSPG secretion have been associated with glial scar formation^[65]^. In summary, the functions of astrocyte-Exos depend on astrocyte activation state: they tend to be neuroprotective under homeostasis but may contribute to injury in inflammatory contexts.

## SECRETED EXOSOMES

SCs play an important role in peripheral nerve regeneration, and their secreted exosomes (SC-Exos) are being investigated as potential contributors to SCI repair. Evidence indicates that SC-Exos modulate post-injury immune responses, protect mitochondria and support axonal regeneration through multiple signaling pathways. SC-Exos mediate angiogenesis and neuroprotection via integrin β1 (ITGB1), supporting local tissue repair^[58]^. They also activate TLR2 on astrocytes and modulate immune responses through NF-κB and PI3K-AKT signaling, biasing inflammatory states towards reparative profiles to reduce secondary tissue damage^[66]^. At the level of cellular homeostasis, SC-Exos enhance autophagy and attenuate apoptosis via the epidermal growth factor receptor (EGFR)-AKT-mTOR pathway, thereby protecting axons from degeneration^[67]^.

Regarding immunomodulation, milk fat globule-epidermal growth factor 8 (MFG-E8) carried by SC-Exos regulates macrophage and microglial activation via suppressor of cytokine signaling 3 (SOCS3)/STAT3 signaling, biasing immune responses towards reparative states^[68]^. SC-Exos also activate AMP-activated protein kinase (AMPK), protecting mitochondria and alleviating oxidative-stress injury^[69]^. Moreover, recent work reports that SC-Exos limit glial scarring - reducing CSPG deposition and glial scar formation - and promote axonal growth, supporting neural regeneration and motor recovery via regulation of the Ras homolog family member A (RhoA)-Rho-associated coiled-coil containing protein kinase (ROCK)-protein tyrosine phosphatase sigma (PTPσ) signaling pathway^[70]^. Taken together, SC-Exos exhibit neurorestorative activities in experimental models by integrating mechanisms that include angiogenesis, immunomodulation, mitochondrial homeostasis and axonal regeneration, thereby highlighting a peripheral-cell source for exosome-based strategies in SCI.

## OEC-EXOS

Research on OEC-Exos in central nervous system repair remains at an early stage; evidence suggests that they modulate the lesion microenvironment and support neural regeneration. Studies report that OEC-Exos bias microglial/macrophage activation towards M2-like states by inhibiting NF-κB and c-Jun signaling^[59]^. In addition, OEC-Exos have been reported to promote neural precursor proliferation and attenuate oxidative stress-induced cytotoxicity^[71]^. Taken together, current data indicate that OEC-Exos support SCI repair via antioxidant and immunomodulatory mechanisms within the microenvironment.

## OLIGO-EXOS

Oligo-Exos support myelin maintenance and neuronal metabolic homeostasis in the central nervous system and also exhibit context-dependent roles in regulating axonal regeneration. Research indicates that the oligodendrocyte membrane protein Nogo-A can be cleaved by β-secretase-1 (BACE1) to generate a 24-kDa C-terminal fragment. This fragment is released in exosomes, bears the Nogo-66 domain on the particle surface and binds the Nogo-66 receptor (NgR1) on neuronal membranes, thereby inhibiting axonal regrowth after injury^[60]^. These observations suggest that Oligo-Exos convey both homeostatic and inhibitory cues during regeneration, helping to delineate guidance boundaries and to limit aberrant circuit rewiring.

In summary, neural cell-derived exosomes play multilevel and complementary roles in SCI repair. Exosomes from different cellular origins have distinct functions in neuroprotection, immunomodulation and regeneration. Future work should elucidate synergistic mechanisms, signaling crosstalk and temporal dynamics among neural exosome populations, and standardize isolation and characterization protocols to advance translational applications in SCI regenerative therapy.

## THERAPEUTIC ROLES AND MECHANISMS OF EXOSOMES FROM ALTERNATIVE SOURCES IN SCI

As research progresses, growing interest has focused on the functions and mechanisms of exosomes from diverse cell types in SCI repair. These exosomes display distinct activities, including immune modulation, angiogenesis, maintenance of tissue homeostasis and neuroprotection, thereby broadening the scope of exosome-mediated repair mechanisms. The scope has expanded beyond traditionally studied sources to include vascular-associated cells, osteoblasts and pluripotent stem cells; central nervous system tissues; animal and plant tissues; and body fluids. The following sections summarize recent advances and mechanistic insights into the roles of these exosomes in SCI repair (representative examples are summarized in Table 4, while a more comprehensive overview is provided in Supplementary Table 4).

**Table 4:** Representative therapeutic effects of Exos isolated from other sources

**Exo sources**	**Effects of Exo-administration**	**Animals**	**Ref.**
ABPC	Induced macrophage polarization from M1 to M2, promoted neural stem cell proliferation and axonal extension, and reduced neuronal apoptosis	Mouse	[72]
CSF	Enhanced Exo uptake by endothelial cells, activation of PI3K/Akt signaling, and improved angiogenesis and neurofunctional recovery	Pig	[73]
Endothelial	Enhanced M2 macrophage polarization, reduced inflammation and ROS levels, downregulated USP13/IκBα/NF-κB signaling	Rat	[74]
EPCs	Increased angiogenesis and tissue repair, reduced monocyte infiltration, improved motor recovery	Mouse	[75]
iPSC	Immune and inflammatory responses were suppressed; neuroprotection was enhanced	Mouse	[76]
Osteoblast	Alleviated ischemia-reperfusion injury via miR-23a-3p/KLF3/CCNL2 regulation	Mouse	[77]
Pericyte	Promoted blood-spinal cord barrier repair and angiogenesis, improved mitochondrial function via miR-210-5p/JAK1-STAT3 activation	Mouse	[78]
Plant	Suppressed inflammation, enhanced neuroprotection, and improved antioxidant capacity	Rat	[79]
Plasma	Enhanced miR-138-5p-mediated epigenetic control, inhibited SOX4 axis, and weakened inflammatory response	Rat	[80]
PRP	Upregulation of neuroprotective factors and reduced inflammatory activity in immune cells	Mouse	[81]
Serum	Strengthened tsRNA-mediated epigenetic regulation, reduced immune inflammation, and altered neuronal survival gene expression	Human	[82]
Spinal cord tissue	Enhanced microglial M1 polarization, upregulated miR-155-5p-mediated epigenetic regulation, activated FOXO3a/NF-κB signaling	Rat	[83]
SVZ	Decreased TNF-α, IL-1β, IL-18, and IL-6 expression, attenuated inflammation, and promoted neuroprotection and tissue repair	Rat	[84]

ABPC: Antler blastema progenitor cell; CSF: cerebrospinal fluid; Endothelial: endothelial cell; EPC: endothelial progenitor cell; iPSC: induced pluripotent stem cell; Plant: plant cell; PRP: platelet-rich plasma; SVZ: subventricular zone; Exos: exosomes; ROS: reactive oxygen species; USP13: ubiquitin-specific protease 13; IκBα: inhibitor of nuclear factor kappa B alpha; NF-κB: nuclear factor kappa B; miR: microRNA; KLF3: Kruppel-like factor 3; CCNL2: cyclin L2; JAK1: Janus kinase 1; STAT3: signal transducer and activator of transcription 3; SOX4: SRY-box transcription factor 4; tsRNA: tRNA-derived small RNA; FOXO3a: forkhead box O3a; TNF-α: tumor necrosis factor alpha; IL: interleukin.

## EXOSOMES FROM OTHER CELLULAR SOURCES

Recent research has increasingly focused on exosomes from diverse cell types in SCI repair. Endothelial cell-derived exosomes have been investigated as contributors. Evidence indicates that exosomes from endothelial progenitor cells (EPCs) accumulate at the injury site after early intravenous administration, promote tissue restoration and angiogenesis, and are associated with improvements in behavioral readouts such as Basso Mouse Scale scores (BMS) scores, inclined-plane performance and pain-related responses^[75]^. Mechanistic studies report that EPC-derived exosomes enriched in miR-222-3p modulate the SOCS3-Janus kinase 2 (JAK2)-STAT3 signaling axis, biasing macrophages toward an M2 anti-inflammatory phenotype; this reshaping of the inflammatory microenvironment alleviates secondary injury and improves motor and electrophysiological function^[85]^. In microvascular endothelial cell-derived exosomes, exosomal Ubiquitin-specific peptidase 13 (USP13) stabilizes Inhibitor of nuclear factor κ-B alpha (IκBα), dampens NF-κB activity, lowers ROS, biases macrophages toward M2 polarization, mitigates neuroinflammation and promotes motor-function recovery^[74]^.

Beyond endothelial cells, pericytes - key components of the microvasculature - also contribute to SCI repair via their exosomes. Pericyte-derived exosomes (Pericyte-Exos) carrying miR-210-5p have been reported to activate Janus kinase 1 (JAK1)-STAT3 signaling, enhance BSCB integrity, improve mitochondrial function and limit lipid peroxidation, supporting endothelial repair and associating with improvements in behavioral recovery^[78]^.

Osteoblast-derived exosomes have been investigated in SCI-related ischemia-reperfusion models. Enriched with miR-23a-3p, they suppress Krüppel-like factor 3 (KLF3)-driven Cyclin L2 (CCNL2) transcription, reduce neuronal apoptosis and mitigate inflammation, and have been associated with reduced neuronal damage in spinal cord ischemia-reperfusion injury (SCIRI)^[77]^.

Exosomes from induced pluripotent stem cells (iPSC-Exos) exhibit immunomodulatory activity: their miR-199b-5p targets hepatocyte growth factor (HGF) and engages PI3K signaling, biasing macrophages toward an M2 phenotype, dampening inflammatory microenvironments and improving neurological recovery^[86]^. Additionally, exosomes from induced pluripotent stem cell (iPSC)-derived NSCs (iPSC-NSC-Exos), enriched in let-7b-5p, inhibit Leucine-rich repeats and immunoglobulin-like domains 3 (LRIG3), modulate microglial and macrophage pyroptosis, reduce IL-1β release and help maintain myelin integrity, thereby supporting neural repair and functional recovery^[87]^.

In summary, exosomes from vascular-associated cells (EPCs, endothelial cells, pericytes), osteoblasts, neurons and iPSC-derived cells exhibit multifaceted activities in modulating inflammation, promoting neuroregeneration and remodeling the lesion microenvironment. Their cell-specific functional profiles provide biological materials and molecular bases for next-generation acellular strategies.

## EXOSOMES FROM OTHER TISSUE SOURCES

Research now examines exosomes from diverse tissues - including central nervous system tissues, animal regenerative tissues and plants - for SCI repair.

Evidence indicates that exosomes from neural tissues (e.g., subventricular zone-derived EVs, SVZ-EVs) reduce levels of inflammatory mediators, limit tissue damage and are associated with improvements in behavioral outcomes in SCI models, supporting neuroimmune modulation and regenerative effects^[84]^.

Exosomes from animal regenerative tissues have been investigated for neurorepair. Exosomes from antler blastema progenitor cells (ABPC-EVs) promote neural stem-cell proliferation and axonal regeneration, bias macrophages towards M2-like states, mitigate inflammation and decrease neuronal apoptosis, with 1.3-fold increases in axonal growth and 2.6-fold improvements in functional recovery reported in that study relative to EVs from BMSCs and NSCs^[72]^.

Plant-derived exosomes are under investigation for modulation of neural microenvironments. Exosomes from Flos Sophorae Immaturus (FSI-Exos), incorporated into polydopamine-modified hyaluronic-acid hydrogel (pDA-gel), form a sustained-release system that reduces oxidative stress, limits ROS accumulation, supports neural regeneration and improves motor and urinary readouts in animal models, providing a rutin-based, exosome-mediated anti-inflammatory and antioxidant approach^[79]^. Similarly, exosomes from Lycium barbarum combined with isoliquiritigenin (ISL) have been reported to modulate local inflammation, promote neurodifferentiation and improve functional recovery^[88]^.

Taken together, exosomes from central nervous system tissues, animal tissues and plants exhibit anti-inflammatory, antioxidant and pro-regenerative activities in SCI models, although context-dependent proinflammatory effects warrant careful evaluation.

## EXOSOMES FROM BODY FLUIDS

Exosomes derived from body fluids - such as plasma, serum and cerebrospinal fluid - are being investigated as contributors to SCI regeneration and diagnosis, given their accessibility and ability to reflect systemic pathological states.

Autologous plasma-derived exosomes (AP-Exos) functionalized with neurotargeting (rabies virus glycoprotein, RVG) and growth-promoting peptides [intracellular LAR peptide (ILP)/intracellular sigma peptide (ISP)] have been reported to enhance delivery to injured spinal regions, improve axonal regeneration and support neural-circuit reconstruction, with associated gains in motor recovery^[89]^.

Platelet-rich plasma (PRP)-derived exosomes (PRP-Exos) exhibit immunomodulatory and anti-apoptotic activity. PRP-Exos loaded with cerebropeptides modulate the TNF-α/IL-10 ratio, decrease BAX and increase BCL-2, thereby reducing apoptosis, supporting tissue preservation and improving Basso-Beattie-Bresnahan (BBB) scores^[81]^. Additional work reports that PRP-EVs increase tight-junction proteins (ZO-1, occludin) and engage AKT and ERK signaling, contributing to blood-BSCB repair^[90]^.

Moreover, melatonin-pretreated plasma exosomes enriched with miR-138-5p target SOX4, bias microglia towards M2-like states, attenuate inflammation and support neural repair^[80]^.

Beyond miRNAs, other noncoding RNAs in plasma exosomes also appear to participate in pathological regulation. For example, tRNA-derived fragment (tRF-41)-enriched serum exosomes from patients with SCI have been reported to modulate Wnt/β-catenin signaling and lower neurotrophic factors, changes associated with astrocyte apoptosis; conversely, reduced miR-429 has been linked to increased neuronal apoptosis via PTEN-PI3K-AKT signaling^[82,91]^.

Taken together, body fluid-derived exosomes - spanning inflammation regulation, vascular regeneration, apoptosis control and neural remodeling - show activity in SCI models and are being explored as candidates for regenerative strategies.

## EXOSOMES IN THE POST- SCI PATHOLOGICAL STATE

Following SCI, exosomes released from injured tissues have been reported to participate in pathological processes and can exert deleterious effects. Studies report that exosomal cargo profiles show marked changes within injured spinal cord tissue. These exosomes can propagate inflammatory signals to adjacent regions and elicit secondary inflammatory responses via connexin 43 (Cx43) hemichannels and dysregulated astrocytic Ca^2+^ dynamics.

Research indicates that such exosomes are enriched in miR-155-5p, which amplifies inflammatory signaling and exacerbates tissue damage by modulating the NF-κB pathway and biasing microglial polarization^[83]^. These observations position pathological exosomes as potential targets and safety considerations in SCI therapy design. Collectively, the data support context-dependent roles of exosomes in SCI - capable of propagating or constraining inflammation - which underscores their relevance to both mechanistic studies and risk assessment^[92]^.

Moreover, exosomes with distinct molecular signatures have been detected in the body fluids of patients with SCI. Specific circRNAs show characteristic expression patterns in these fluids^[93]^. Notably, the composition of body fluid-derived exosomes appears to be influenced by individual physiological states. These findings suggest non-invasive, longitudinal avenues for monitoring disease progression and indicate potential utility as biomarkers for SCI subtyping and early screening.

In ageing SCI models, exosomes have been reported to carry elevated proinflammatory molecules, provoking secondary inflammatory responses in remote brain regions^[23,94]^. Studies of sex differences further report that exosomes from males more frequently elicit proinflammatory responses, whereas those from females tend to show relatively anti-inflammatory properties^[95]^. These findings point to individualized exosomal mechanisms in SCI and inform the development of precision-oriented therapeutic strategies.

In summary, body fluid-derived exosomes show promise as biomarker candidates for early diagnosis, prognostic assessment and personalized treatment stratification in SCI. They may help integrate pathological subtyping with targeted interventions as the field advances.

## IMPACT OF INJURY MODELS AND ADMINISTRATION ROUTES ON THERAPEUTIC OUTCOMES

Interpretation and comparison of preclinical studies evaluating exosome-based therapies for SCI are complicated by substantial heterogeneity in experimental design. Animal models vary widely in injury type and severity, including contusion, compression, transection, and ischemia-reperfusion paradigms, each of which recapitulates distinct pathological features of human SCI. These differences critically influence inflammatory dynamics, BSCB disruption, axonal degeneration, and regenerative potential, thereby shaping the observed therapeutic efficacy of exosome administration.

In parallel, administration routes - including intravenous, intrathecal, and local implantation - introduce additional variables affecting biodistribution, target-cell engagement, and safety profiles. Systemic intravenous delivery is often favored for immunomodulation and peripheral immune reprogramming but may suffer from off-target sequestration by the reticuloendothelial system. In contrast, intrathecal or intraparenchymal delivery enhances local bioavailability and may be more effective for BSCB repair and axonal regeneration, albeit with increased procedural complexity.

Despite these variations, few studies provide direct comparisons across models or delivery routes, limiting cross-study reproducibility. Standardized reporting of injury parameters, dosing regimens, timing of administration, and functional endpoints would greatly facilitate mechanistic interpretation and translational assessment. Future investigations should incorporate head-to-head comparisons to define optimal delivery strategies tailored to specific therapeutic goals.

## ENHANCING THE THERAPEUTIC EFFICACY OF EXOSOMES FOR SCI THROUGH CELL PRECONDITIONING

The therapeutic potential of exosomes in repairing SCI is increasingly investigated; however, their bioactivity is often constrained by the physiological state of the parent cells. To enhance neuroprotective and regenerative capacities, researchers are exploring cell preconditioning strategies to modulate exosome functionality. Preconditioned exosomes are those released by source cells after exposure to specific stimuli - such as hypoxia, inflammatory cues, pharmacological agents or genetic modulation - resulting in altered molecular cargo and signaling relative to unstimulated exosomes, with reported functional gains in injury models. This strategy aims to modulate the inductive microenvironment and reshape exosomal cargo profiles (e.g., miRNAs, proteins and lipids), supporting neuronal survival, axonal regeneration and restoration of immune homeostasis (representative examples are summarized in Table 5, while a more comprehensive overview is provided in Supplementary Table 5).

**Table 5:** Representative preconditioning strategies reported to enhance the therapeutic effects of Exos in spinal cord injury

**Exo sources**	**Preconditioning method**	**Effects of Exo-administration**	**Animals**	**Ref.**
hTERT-MSC	Hypoxia	Inflammatory cytokines decreased, neuronal apoptosis reduced; miR-511-3p regulated TRAF6/S1PR3/NF-κB pathway	Mouse	[96]
UC-MSC	IL-4	M1→M2 macrophage polarization; inflammation and apoptosis ↓; miR-21-5p targeted PDCD4	Mouse	[97]
Spinal cord tissue	LPS	LPS-EVs activated astrocytes, enhanced inflammatory signaling and hemichannel permeability	Mouse	[92]
MSC	TGF-β1	Enhanced neuroprotection, suppressed inflammation, promoted axonal growth	Mouse	[98]
Plasma	Melatonin	miR-138-5p epigenetic regulation ↑; SOX4 signaling inhibited	Rat	[80]
Microglia	Resveratrol	Neuroprotection ↑, apoptosis ↓, PI3K pathway activated	Rat	[99]
Neuron	Rg1	Microglial M2 ↑, ROS ↓; MYCBP2/S100A9 axis reduced oxidative stress and enhanced repair	Mouse	[100]
UC-MSC	BME	Cytokines ↓, neuronal survival ↑; GFAP/C3 ↓; Yap1 pathway regulated, bladder function improved	Mouse	[101]
UC-MSC	Curcumin	M1→M2 macrophage shift, inflammation ↓, remyelination and regeneration ↑; efficient curcumin release	Mouse	[102]
UC-MSC	Micro-electrical field stimulation	Autophagy ↑, apoptosis ↓; lncRNA-MALAT1/miR-22-3p activated SIRT1/AMPK pathway	Rat	[103]

↑ Indicates upregulation; ↓ indicates downregulation; → indicates a change in the direction of the phenotypic outcome. hTERT-MSC: Human telomerase reverse transcriptase-immortalized mesenchymal stem cell; UC-MSC: umbilical cord mesenchymal stem cell; ADSC: adipose-derived mesenchymal stem cell; BMSC: bone marrow mesenchymal stem cell; MSC: mesenchymal stem cell; NSC: neural stem cell; Exos: exosomes; IL-4: interleukin 4; LPS: lipopolysaccharide; EVs: extracellular vesicles; TGF-β1: transforming growth factor beta 1; miR: microRNA; TRAF6: tumor necrosis factor receptor-associated factor 6; S1PR3: sphingosine-1-phosphate receptor 3; NF-κB: nuclear factor kappa B; PDCD4: programmed cell death 4; PI3K: phosphoinositide 3-kinase; ROS: reactive oxygen species; MYCBP2: MYC binding protein 2; S100A9: S100 calcium-binding protein A9; BME: brain microvascular endothelial; GFAP: glial fibrillary acidic protein; C3: complement component 3; YAP1: Yes-associated protein 1; lncRNA: long non-coding RNA; MALAT1: metastasis-associated lung adenocarcinoma transcript 1; SIRT1: sirtuin 1; AMPK: AMP-activated protein kinase.

Among preconditioning methods, hypoxic preconditioning is one of the more widely studied approaches. A hypoxic environment upregulates HIF-1α in source cells, thereby altering exosome cargo and enriching pro-angiogenic and anti-apoptotic molecules. For example, exosomes from hypoxia-treated human umbilical-cord MSCs increase angiogenic capacity: endothelial or stromal cells stimulated with hypoxia-preconditioned exosomes (HPC-Exos) show improved migration and tube formation *in vitro*; in a spinal cord transection model, administration of these exosomes promoted angiogenesis, attenuated inflammation and improved neurological function^[104]^. Another study reported that hypoxia-preconditioned adipose-derived MSC (ADSC)-derived exosomes (ADSC-Exos) were enriched with lncRNA Gm37494, which biased microglial polarization from M1-like to M2-like states by inhibiting miR-130b-3p and upregulating peroxisome proliferator-activated receptor γ (PPARγ), thereby reducing inflammation and supporting functional recovery after SCI^[105]^. Further work showed that hypoxic ADSC-Exos reduced neuronal apoptosis in an oxygen-glucose deprivation/reperfusion (OGD/R) model and decreased lesion size while improving hindlimb motor function in a rat SCI model^[106]^. Collectively, these findings support the view that hypoxic preconditioning, by mimicking post-injury cues, endows exosomes with enhanced pro-regenerative activity, offering new perspectives for SCI therapy.

Besides hypoxia, inflammatory-cytokine preconditioning has been explored to enhance the immunomodulatory capacity of exosomes. During the acute phase of SCI, excessive immune activation often leads to secondary neural damage. Thus, preconditioning source cells with inflammatory cues to generate exosomes enriched in anti-inflammatory cargo is being investigated as a potential approach. For example, exosomes from IL-4-preconditioned human umbilical-cord MSCs (IL-4-hUC-MSC-Exos) are rich in miR-21-5p, which biases macrophage polarization from M1-like to M2-like states by targeting programmed cell death 4 (PDCD4). This reduces post-SCI inflammation and neuronal apoptosis, with reported improvements in motor recovery^[97]^. In contrast, proinflammatory stimuli can confer different biological properties. Exosomes released from LPS-stimulated spinal cord organotypic slices (LPS-exosomes) can propagate inflammatory responses via Cx43 hemichannel-mediated signaling, eliciting Ca^2+^ dysregulation and activation in unstimulated astrocytes^[92]^. Inflammatory preconditioning can also regulate astrocyte behavior by altering the miRNA content of microglial exosomes: exosomes from LPS-activated microglia show downregulation of miR-145-5p, increasing SMAD family member 3 (SMAD3) expression and driving excessive astrocyte proliferation; transfection with a miR-145-5p mimic attenuates this effect^[107]^. These data highlight the role of inflammatory signaling in modulating the microglia-astrocyte axis and suggest that tuning exosomal miRNA composition may enable indirect intervention in glial scar formation. Taken together, inflammatory signals are not only triggers of pathological damage but can also be leveraged for exosomal “immune reprogramming”, supporting more precise modulation of SCI inflammatory cascades through control of cargo composition and targeting.

In pharmacological preconditioning, researchers apply specific drugs or compounds to modulate source-cell metabolic pathways, thereby reshaping exosome cargo. Melatonin, a well-characterized preconditioning agent with antioxidant and anti-inflammatory properties, is commonly used in stem-cell studies. Exosomes from melatonin-treated microglia enhanced anti-inflammatory activity in SCI models, increasing microglial and macrophage phagocytosis of myelin debris and helping to preserve BSCB integrity. Neurons and oligodendrocyte precursor cells were biased towards reparative states, with increased remyelination and axonal growth, with reported improvements in therapeutic outcomes^[108]^. Similarly, resveratrol-preconditioned exosomes showed enhanced neuroprotective activity, with reduced neuronal apoptosis and engagement of PI3K signaling^[99]^. Additional studies report that preconditioning agents such as ginsenoside Ginsenoside Rg1 (Rg1) and β-mercaptoethanol (BME) improve the neuroprotective activity of exosomes by modulating cellular oxidative stress or epigenetic pathways^[100,101]^. Together, these studies suggest a pharmacological route to functionally enhanced exosomes through modulation of donor-cell states.

Physical and chemical preconditioning also provide additional means of regulating exosome function. It has been reported that micro-electric-field stimulation induces MSCs to release exosomes enriched with lncRNA metastasis-associated lung adenocarcinoma transcript 1 (MALAT1), activating the MALAT1/miR-22-3p/Sirtuin 1 (SIRT1)/AMPK axis, enhancing autophagy, reducing apoptosis and improving motor function after SCI^[103]^.

In summary, preconditioning strategies can enhance exosome function through multiple pathways and constitute an important branch of exosome engineering. Future work could combine multimodal preconditioning to precisely tune exosomal cargo composition and evaluate long-term safety and stability. In addition, standardization of preconditioning parameters - such as stimulus type, concentration, duration and cell source - will require systematic optimization to advance clinical translation in SCI therapy.

## EXOSOMES AS DELIVERY PLATFORMS FOR SCI TREATMENT

Natural exosomes face limitations in loading capacity, *in vivo* stability and BSCB penetration, which hinder effective targeted delivery and controlled release. To address these challenges, researchers have developed engineering strategies to enhance exosome functionality and controllability without compromising biocompatibility or signaling properties. In this Review, we use the term engineered exosomes to refer to vesicles modified by physical, chemical or surface-conjugation methods^[109]^ [Table 6].

**Table 6:** Exo as a delivery platform for spinal cord injury treatment

**Exo sources**	**Loading protocol**	**Loaded components**	**Effects of EV administration**	**Targeted cells**	**Route**	**Ref.**
Autologous plasma	Surface anchoring loading	RVG, ILP, and ISP peptides (anchored via CP05 on EV surface)	Promotes neuronal accumulation at lesion; reduces GFAP; restores 5-HT and CST axons; improves BMS, gait, and MEP	Primarily neurons (minor uptake by astrocytes and microglia)	Tail vein injection	[89]
Autologous plasma	Electroporation	miR-138-5p mimics or inhibitor	Improves motor and MRI outcomes; reduces inflammation; miR-138-5p/SOX4 axis drives M2 polarization and remyelination	Microglia	Not reported	[80]
HUC-MSC	Ultrasound	Quercetin	Inhibits TLR4/NF-κB and JAK2/STAT3 pathways; suppresses A1 astrocytes and M1 microglia; enhances axonal growth and functional recovery	Microglial; astrocyte; neuron	Tail vein injection	[110]
HUC-MSC	Electroporation	CRISPR/Cas9 plasmid; CAQK targeting peptide	Targets injured site; lowers TNF-α, IL-1β, IL-6 via NF-κB inhibition; promotes M2 macrophage polarization and motor improvement	Immune cells within lesion site, mainly macrophages (including T cells and neutrophils)	Tail vein injection	[111]
HUC-MSC	Genetic engineering surface modification	Surface ligand RGD peptide; endogenous miR-501-5p	Binds vascular endothelium; decreases BSCB permeability via miR-501-5p-MLCK axis; upregulates tight junctions; restores motor function	Neovascular endothelial cells (CD105^+^, integrin αvβ3^+^)	Intranasal administration	[112]
HUC-MSC	Co-incubation/Ultrasound	AM1241 (CB2 receptor agonist)	Enhances motor coordination; suppresses GFAP and Iba-1 via lowering IL-1β/IL-6/TNF-α; activates Nrf2/HO-1; promotes antioxidation and antiapoptosis	Neural stem cells (*in vitro*); astrocytes, microglia, and neurons (*in vivo*)	Intrathecal injection	[113]
iNSC	Surface modification	CCL2-siRNA; CAQK targeting peptide	Accumulates in reactive astrocytes; suppresses CCL2 and oxidative stress; reduces apoptosis; promotes axon regrowth and remyelination	Reactive astrocytes (CSPG^+^); microglia/macrophages; neurons	Tail vein injection	[114]
M2-macrophage	Ultrasound	Berberine	Improves local retention; inhibits inflammation; drives M1 to M2 microglia/macrophage transition; reduces neuronal apoptosis and lesion volume	Microglia/macrophages; neurons	Tail vein injection	[115]
MUC-MSC	Co-incubation	Curcumin	Reduces inflammation and shifts macrophage/microglia from M1 to M2 phenotype; promotes axonal regeneration and remyelination; improves BBB and motor function	Microglial/macrophage; neuron; oligodendrocyte	Local injection	[102]
NSC	Ultrasound	FTY720 (Fingolimod)	Protects BSCB integrity; reduces apoptosis; restores neuronal morphology; improves BBB and electrophysiology via PTEN/AKT signaling	Spinal microvascular endothelial cells; neurons	Tail vein injection	[61]
NSC	Surface modification	Surface ligands: CAQK + Angiopep-2; no external drug/nucleic acid payload	Enhances lesion targeting; reduces inflammation and gliosis; promotes neurofiber regeneration; improves BMS and MEP scores *vs*. unmodified EVs	Endothelial cells; microglia/macrophages; neurons	Tail vein injection	[116]
PRP	Ultrasound	Cerebrolysin (neurotrophic peptide mixture)	Restores immune balance; inhibits apoptosis; reduces lesion size; markedly improves BBB score	Not reported	Tail vein injection	[81]
PRP	Ultrasound	Dexamethasone	Reduces inflammation; decreases lesion and lymphocyte infiltration; elevates BBB locomotor score	Not reported	Tail vein injection	[117]
RBM-MSC	Electroporation	CTGF-targeted siRNA	Downregulates CTGF and fibrosis-related proteins; reduces inflammation and apoptosis; elevates BDNF/TGF-β1; improves locomotion and bladder function	Astrocytes, DRG neurons, macrophages (*in vitro*); spinal tissue-related cells (*in vivo*)	Tail vein injection	[118]
RBM-MSC	Transfection	miR-494 mimics	Suppresses TNF-α and IL-6; promotes IL-8/IL-10 upregulation; reduces neuronal apoptosis; enhances BBB and electrophysiology	Neurons (DRG); macrophages	Tail vein injection	[119]

HUC-MSC: Human umbilical cord mesenchymal stem cell; iNSC: induced neural stem cell; MUC-MSC: mouse umbilical cord mesenchymal stem cell; NSC: neural stem cell; PRP: platelet-rich plasma; RBM-MSC: rat bone marrow mesenchymal stem cell; DRG: dorsal root ganglion; Exo: exosome; EV: extracellular vesicle; RVG: rabies virus glycoprotein; ILP: ischemic lesion-targeting peptide; ISP: intracellular sigma peptide; GFAP: glial fibrillary acidic protein; 5-HT: 5-hydroxytryptamine; CST: corticospinal tract; BMS: Basso Mouse Scale; MEP: motor evoked potential; MRI: magnetic resonance imaging; miR: microRNA; SOX4: SRY-box transcription factor 4; TLR4: Toll-like receptor 4; NF-κB: nuclear factor kappa B; JAK2: Janus kinase 2; STAT3: signal transducer and activator of transcription 3; CRISPR: clustered regularly interspaced short palindromic repeats; TNF-α: tumor necrosis factor alpha; IL: interleukin; RGD: arginine-glycine-aspartate; BSCB: blood-spinal cord barrier; MLCK: myosin light chain kinase; CD105: cluster of differentiation 105; Nrf2: nuclear factor erythroid 2-related factor 2; HO-1: heme oxygenase 1; CCL2: C-C motif chemokine ligand 2; siRNA: small interfering RNA; CSPG: chondroitin sulfate proteoglycan; BBB: Basso, Beattie, and Bresnahan locomotor rating scale; PTEN: phosphatase and tensin homolog; CTGF: connective tissue growth factor; BDNF: brain-derived neurotrophic factor.

Compared with natural exosomes, engineered exosomes offer advantages in drug delivery, cargo loading and targeting^[120]^. They can improve drug stability and bioavailability, and enable preferential accumulation in target tissues or cells, thereby enhancing therapeutic efficacy while reducing off-target effects. Current work in SCI largely centers on two strategy classes: drug-loaded exosomes and surface-modified exosomes. This section summarizes advances in these directions, focusing on structural and functional engineering of the vesicles themselves and excluding genetic modification of donor cells, preconditioning and hybrid material systems.

Drug-loaded exosomes are created using physical or chemical methods to load small molecules, nucleic acids, and proteins into the lumen or onto the membrane for precise delivery and combination therapy^[120]^. Common approaches include passive incubation, electroporation, sonication and freeze-thaw cycling^[121]^. Passive incubation is simple and preserves membrane integrity but typically yields limited loading efficiency^[120]^. Electroporation can increase encapsulation efficiency, albeit with risks of membrane perturbation and cargo aggregation^[122]^. Sonication and freeze-thaw methods offer a practical balance between loading efficiency and structural stability, and have been adopted across multiple studies^[123]^.

Engineered drug-loaded exosomes typically act via two complementary mechanisms. First, vesicles retain intrinsic anti-inflammatory, anti-apoptotic and pro-regenerative activities; second, the loaded drugs potentiate modulation of specific signaling pathways, strengthening neuroprotection and functional recovery. For example, PRP-Exos loaded with Cerebrolysin and given intravenously in SCI models improved motor scores and reduced neuronal apoptosis^[81]^. Similarly, NSC-Exos loaded with FTY720 (fingolimod) limited neuronal apoptosis via the PTEN-AKT axis and supported axonal regeneration and functional recovery^[61]^. In addition, human umbilical-cord MSC-derived exosomes (hUCMSC-Exos) have been used to deliver flavonoids (e.g., quercetin), attenuating inflammatory responses, reducing glia-related pathology and improving motor function^[110]^.

For nucleic-acid and protein cargoes, non-genetic engineering approaches are under investigation. Physical or chemical methods (e.g., electroporation, conjugation) enable loading of exogenous miRNA mimics, small interfering RNAs (siRNAs) or proteins to modulate defined pathways. For instance, miR-21-5p mimics were electroporated into human adipose stem cell-derived exosomes^[124]^. In a separate study, MSC-derived exosomes electroporated with PTEN siRNA, delivered intranasally in a rat complete-SCI model, improved motor function and promoted axonal regeneration^[125]^. Likewise, connective tissue growth factor (CTGF)-targeting siRNA-loaded MSC-Exos suppressed CTGF and inflammatory mediators (TNF-α, IL-1β) after intravenous dosing and improved motor outcomes^[118]^. Furthermore, chemical conjugation of the neurotropic peptide RVG and growth-promoting peptides ILP/ISP to autologous plasma exosomes using the CD63-anchoring peptide CP05 enabled targeted enrichment at the injury site, enhancing axonal growth across glial scars and improving motor function^[89]^.

Surface-modified exosomes focus on membrane engineering to improve targeting and *in vivo* stability. Targeting strategies tune physicochemical properties and administration routes to achieve preferential distribution in injured spinal cord. *In vivo* behavior relates to vesicle size, surface charge, membrane-protein conformation and injection route. Intrathecal injection provides direct delivery to the central nervous system, bypassing peripheral clearance mechanisms; however, it carries risks of secondary injury and infection, and repeated dosing can be challenging. By contrast, intravenous injection may be suited to systemic immunomodulation or suppression of chronic inflammation, is straightforward and permits repeated dosing^[126]^. Because natural exosomes are rapidly cleared by the mononuclear-phagocyte system, tissue retention is limited. To address this, researchers decorate exosomal membranes with ligand peptides, antibodies or polymers to confer cell/tissue recognition. Common strategies include covalent linkage, membrane fusion and lipid insertion - notably, post-insertion of 1,2-Distearoyl-sn-glycero-3-phosphoethanolamine (DSPE)-polyethylene glycol (PEG) lipids - which can preserve membrane integrity^[127]^. CD47 functions as a “don’t-eat-me” signal, prolonging circulation; increasing CD47 display on engineered exosomes has been reported to enhance *in vivo* retention^[128]^. In SCI models, CAQK peptide (Cys-Ala-Gln-Lys) (CAQK)-modified NSC-Exos selectively accumulated at lesions, reducing inflammation and oxidative stress^[114]^. Similarly, Angiopep-2-modified engineered exosomes showed enhanced the central nervous system barrier penetration and lesion enrichment, supporting remyelination and neurological recovery^[108]^.

Additionally, PEG modification can reduce non-specific interactions with plasma components, extend circulation time and improve delivery after intravenous administration^[127]^. Together, these studies suggest that surface modification can enhance targeting and *in vivo* stability, supporting spatiotemporal control of delivery while preserving bioactivity. However, challenges remain in maintaining native membrane-protein conformation and activity and in ensuring batch-to-batch consistency. Future work should prioritize site-specific, stoichiometrically defined conjugation systems and robust quality-control standards to improve the reproducibility and safety of engineered exosomes.

A dual-targeting strategy - combining peptide-based tissue homing with receptor-mediated cellular uptake - enables two-level precision. Peptides such as CAQK and Angiopep-2 promote lesion-site accumulation, whereas receptor pathways [e.g., low-density lipoprotein receptor-related protein 1 (LRP1), neural cell adhesion molecule (NCAM)] increase cellular internalization, thereby enhancing overall targeting performance^[116,129]^. These approaches may not only facilitate passage across BSCB but also provide routes toward spatiotemporally controlled neural repair.

In summary, engineered exosomes - via drug loading, surface modification and targeted delivery - can address multiple limitations of natural vesicles and are being explored for regenerative SCI therapy. With advances in chemical biology and nanofabrication, higher-precision, more controllable exosome-engineering systems may enable safer and more efficient platforms for nervous-system repair. These developments also provide a foundation for subsequent studies on exosome-biomaterial hybrid systems.

## EXOSOMES INTEGRATED WITH BIOMATERIALS FOR SCI TREATMENT

Exosomes have been widely investigated for SCI repair owing to their biocompatibility and intercellular signaling capabilities. However, stand-alone use faces limitations - including limited *in vivo* residence, suboptimal tissue penetration and uncontrolled release kinetics - that can restrict therapeutic efficacy. To address these issues, exosomes are increasingly combined with biomaterials to construct controlled-delivery systems. This approach aims to prolong activity, enable local sustained therapy and enhance tissue-regeneration capacity^[130]^. Such systems provide physical support and biochemical protection and, by mimicking ECM-like features, can promote neurite outgrowth and cell adhesion, thereby establishing more favorable conditions for SCI repair^[131]^ [Table 7].

**Table 7:** Exosomes combined with biomaterials for treating spinal cord injury

**Exo sources**	**Biomaterial system**	**Administration route**	**Effects of Exo-administration**	**Animals**	**Ref.**
3D-MSCs	GelMA hydrogel microneedle patch loaded with exosomes	Local implantation of microneedle patch at lesion site (intraoperative implantation)	Promotes M1→M2 polarization, reduces inflammation, and enhances neurogenesis, myelination, and angiogenesis	Rat/clip-compression	[132]
ADSCs	Collagen-fibrin hydrogel loaded with exosomes	Local injection/implantation	Induces M1→M2 shift, promotes NSC differentiation into neurons/oligodendrocytes, and facilitates axonal regeneration	Rat/contusion	[133]
ADSCs	F127-PCE hydrogel loaded with exosomes	Local injection	Recruits endogenous NSCs, inhibits glial scarring, and promotes axonal regrowth and tissue bridging	Rat/complete transection	[134]
ADSCs	Injectable poly-L-lysine hydrogel + pH-responsive aminoguanidine nanoparticles + exosomes	Local implantation of hydrogel	Enhances neuroregeneration and remyelination, reduces astrocyte activation, inflammation, and apoptosis	Rat/complete transection	[135]
ADSCs	ROS-responsive dual dynamic hydrogel loaded with exosomes	Local injection	Inhibits NLRP3/Caspase-1, activates Nrf2/HO-1 antioxidant signaling, and reduces oxidative damage and glial scars	Rat/complete Transection	[136]
ADSCs	Silk fibroin hydrogel encapsulating engineered exosomes (RNAi-Tim3)	Local injection	Promotes NSC survival and neuronal differentiation, enhances remyelination, and limits astrocyte formation	Mouse/compression	[137]
BMSCs	GelMA hydrogel loaded with exosomes	Local injection	Induces M2 microglia, inhibits NF-κB, activates PTEN/PI3K/AKT/mTOR, promoting axon growth and remyelination.	Rat/contusion	[138]
BMSCs	GelMA-PPy dual-network conductive hydrogel loaded with exosomes	Local implantation	Promotes neurofilament regeneration and increases NF protein expression	Mouse/right-side hemisection	[139]
BMSCs	Decellularized porcine spinal cord matrix hydrogel loaded with exosomes	Local injection	Guides axon alignment, reduces inflammation, oxidative stress, and apoptosis, and limits glial scarring	Mouse/complete transection	[140]
cBMSCs	Cationized gelatin loaded with exosomes	Local implantation	Suppresses inflammation and fibrosis, promotes remyelination and axonal repair	Rat/contusion	[141]
Cortical neurons differentiated from human iPSCs	hUC-MSC decellularized matrix hydrogel loaded with exosomes	Local injection	Reduces oxidative stress and inflammation, enhances neural tissue repair	Rat/contusion	[131]
CBD-Lamp2b/miR-21	Functionalized type I collagen scaffold loaded with engineered exosomes	Local implantation	Activates Nrf2/HO-1 pathway, mitigates oxidative stress, and promotes neurovascular repair	Rat/complete Transection	[142]
3D-hUC-MSC-Exos	Hyaluronic acid-phenylboronic acid hydrogel + 3D-MSC Exo + dexamethasone	Local injection	Inhibits TLR4/MyD88/NF-κB signaling, decreases scarring, and promotes axonal regeneration	Rat/complete Transection	[143]
plant-derived exosomes, so-Exos	Dopamine-modified hyaluronic acid hydrogel loaded with exosomes	Local implantation	Promotes M1→M2 shift, reduces inflammation, induces NSC differentiation, and limits astrocyte proliferation	Rat/complete transection	[79]
GNA12/GNA13-overexpressing macrophages	Chitosan/β-glycerophosphate thermosensitive nasal spray hydrogel loaded with exosomes	Intranasal administration	Reduces inflammation and oxidative stress, promotes neurogenesis and angiogenesis	Mouse/contusion	[144]
hUC-MSCs	Coaxially printed core-shell hydrogel microfibers	Local implantation	Improves immune microenvironment, protects residual axons, and preserves vasculature/myelin	Rat/complete Transection	[145]
hUC-MSCs	GelMA hydrogel loaded with exosomes + Fe_3_O_4_@BaTiO_3_ nanoparticles	Local implantation	Alleviates oxidative stress and inflammation, enhances axon and myelin regeneration	Rat right-side hemisection SCI	[146]
hUC-MSCs	Linear ordered collagen scaffold with bispecific peptide-anchored exosomes	Local implantation	Reduces inflammatory response	Rat/complete transection	[147]
hUC-MSCs	Gelfoam gelatin sponge loaded with exosomes	Local implantation	Inhibits inflammation and glial scar formation	Rat right-side hemisection SCI	[148]
hUC-MSCs	PLGA-PEG-PLGA hydrogel loaded with engineered miR-138-5p exosomes	Local injection	Reduces inflammation and oxidative stress, promotes axonal regeneration	Rat clip-/compression	[149]
hMSCs	ROS-responsive PVA-TPA hydrogel scaffold loaded with exosomes	Local implantation	Reduces oxidative stress, apoptosis, and glial scarring	Rat/complete transection	[150]
hMSCs	GelMA microneedle patch loaded with exosomes	Subdural surface attachment	Drives M1→M2c and A1→A2 transitions, enhances phagocytosis, glutamate clearance, and synaptic repair	Rat/contusion	[151]
hPAMSCs	Laminin peptide-modified hyaluronic acid hydrogel loaded with exosomes	Local implantation	Inhibits inflammation, activates PI3K/AKT/mTOR, promotes neurogenesis, axon growth, and remyelination	Rat/complete transection	[130]
hUC-MSCs	Fibrin glue loaded with exosomes	Local injection	Promotes endogenous NSC differentiation, neurite elongation, and remyelination	Rat/complete transection	[152]
hUSCs	Hydrogel loaded with exosomes	Local injection and surface coverage of injured area	Reduces apoptosis and glial scars, promotes neurogenesis	Mouse/contusion	[152]
Goji berry	GelMA hydrogel scaffold loaded with exosomes	Local implantation	Suppresses glial activation, reduces oxidative damage, and enhances neural regeneration	Rat/complete Transection	[88]
M2 macrophages	Exosome-loaded Mn-doped Prussian blue nanoparticles	Local injection	Induces M1→M2 shift, reduces neuronal death, activates NSCs, and promotes axon/myelin regeneration	Mouse/contusion	[153]
M2 microglia	Tannic acid/polypyrrole conductive hydrogel loaded with exosomes	Local site implantation	Reduces oxidative stress and apoptosis, promotes M1→M2 transition, remyelination, and angiogenesis	Rat/complete Transection	[154]
MSCs	GelMA helical guidance scaffold + IKVAV gradient covalent modification + exosomes	Local implantation	Attenuates inflammation and oxidative stress, enhances neuronal and myelin regeneration	Rat/complete transection	[155]
MSCs	Oxidized hyaluronic acid-aniline tetramer/gelatin + exosomes (NgR) + NT3	Local implantation	Suppresses Tim3-related inflammation, promotes axonal repair, angiogenesis, and barrier protein expression	Rat/hemisection	[156]
NSCs	IGF-1 bioactive supramolecular nanofiber hydrogel loaded with exosomes	Local implantation	Activates PI3K/AKT signaling to promote angiogenesis	Rat/complete transection	[157]
pCSF	GelMA/HA-NB/LAP hydrogel loaded with exosomes	Local surface coverage	Activates AKT signaling, promotes M1→M2 polarization, reduces inflammation, and enhances neuroregeneration	Mouse/contusion	[73]
SCs	PLGA-PEO electrospun nanofibers@methylprednisolone + HA hydrogel + exosomes	Coverage of injury surface	Induces M1→M2 macrophage shift, reduces neuronal apoptosis, and promotes neurogenesis	Rat/contusion	[158]
hUC-MSC-sEVs	Functionalized hydrogel loaded with exosomes	Local implantation	Promotes angiogenesis, enhances endothelial proliferation and axonal regeneration	Rat/complete transection	[159]

Exo: Exosome; 3D-MSC: three-dimensional cultured mesenchymal stem cell; ADSC: adipose-derived mesenchymal stem cell; BMSC: bone marrow mesenchymal stem cell; cBMSC: cortical bone-derived mesenchymal stem cell; 3D-hUC-MSC: three-dimensional cultured human umbilical cord mesenchymal stem cell; hUC-MSC: human umbilical cord mesenchymal stem cell; hMSC: human mesenchymal stem cell; hPAMSC: human placental amniotic mesenchymal stem cell; hUSC: human urine-derived stem cell; MSC: mesenchymal stem cell; NSC: neural stem cell; SC: Schwann cell; pCSF: porcine cerebrospinal fluid; GelMA: gelatin methacryloyl; F127-PCE: Pluronic F127-poly(ε-caprolactone)-poly(ethylene glycol); ROS: reactive oxygen species; NLRP3: NOD-like receptor family pyrin domain containing 3; Nrf2: nuclear factor erythroid 2-related factor 2; HO-1: heme oxygenase 1; RNAi: RNA interference; NF-κB: nuclear factor kappa B; PTEN: phosphatase and tensin homolog; PI3K: phosphoinositide 3-kinase; AKT: protein kinase B; mTOR: mechanistic target of rapamycin; PPy: polypyrrole; PLGA: poly(lactic-co-glycolic acid); PEG: polyethylene glycol; PVA: poly(vinyl alcohol); TPA: triphenylamine; IKVAV: isoleucine-lysine-valine-alanine-valine peptide; NgR: Nogo receptor; NT3: neurotrophin-3; IGF-1: insulin-like growth factor 1; HA: hyaluronic acid; HA-NB: hyaluronic acid-norbornene; LAP: lithium phenyl-2,4,6-trimethylbenzoylphosphinate; PEO: polyethylene oxide; sEVs: small extracellular vesicles; iPSC-neuron: cortical neuron differentiated from human induced pluripotent stem cell; HEK293T: human embryonic kidney 293T cell; So-Exo (Plant): plant-derived exosome (sophora japonica-derived).

Such systems provide physical support and biochemical protection and, by mimicking ECM-like features, can promote neurite outgrowth and cell adhesion, thereby establishing more favorable conditions for SCI repair^[160]^. For example, gelatin-based conductive hydrogels supported sustained release of MSC-Exos at the lesion for two weeks, promoting axonal regeneration and myelination in experimental models^[139]^. Carrier systems based on natural polysaccharides, such as hyaluronic acid or chitosan, have likewise shown favorable performance in maintaining exosome stability and local concentration, reflecting good biocompatibility and biodegradability^[161]^.

In our previous work, we identified genetically programmed macrophage-derived exosomes (G12/G13-Mφ-Exos) with immunomodulatory activity that bias macrophages toward an M2c anti-inflammatory state and induce a neuroprotective phenotype in astrocytes^[144,162]^. For delivery, we developed an intranasal, thermosensitive chitosan-hydrogel platform. The formulation gels at physiological temperature, enabling *in situ* fixation and stable retention on the nasal mucosa. Relative to conventional routes, this platform extended local residence time and release duration, yielding a sustained release profile, improving targeted-delivery efficiency and providing material support for longer-acting exosome interventions in central nervous system models.

Beyond natural polysaccharides, self-assembling peptides (SAPs) and protein-based biomaterials offer distinctive features for exosome immobilization and directional release. SAPs such as the self-assembling peptide RADA16-I (Ac-(RADA)_4_-CONH_2_) form three-dimensional nanofiber networks via hydrogen bonding and electrostatic interactions, providing an ECM-mimetic milieu for exosomes. Studies report that RADA16-I-based gels attenuate local inflammation, support angiogenesis and increase axon-related marker expression, thereby supporting spinal-cord tissue repair^[163]^. Similarly, collagen/silk-fibroin composite hydrogels incorporating cell-adhesive motifs [e.g., the RGD (Arg-Gly-Asp) peptide motif, the IKVAV (Ile-Lys-Val-Ala-Val) peptide sequence] support neuronal adhesion and synapse formation, with associated improvements in motor function after exosome loading. For example, collagen-I scaffolds functionalized with biospecific peptides anchor and sustain the release of MSC-Exos or drugs, supporting NSC migration and motor recovery^[147]^. Silk-fibroin composite hydrogels loaded with engineered exosomes have also shown improvements in tissue repair and functional recovery in SCI models^[137]^.

A key design consideration for exosome-biomaterial systems is tight control of release kinetics. Broadly, hydrogel-based release can be organized into three modes: physical entrapment for sustained release, covalent or affinity tethering for stabilization and stimuli-responsive release. Tuning crosslink density and pore size can approximate near-linear profiles. Using degradable linkers to tether exosomes to scaffolds can limit initial burst. In addition, peptide ligands displayed on the material surface can engage cell-surface receptors, enhancing targeted uptake and neurite outgrowth^[164]^. These designs aim to align release kinetics with the timescale of neural regeneration and to help maintain an effective local concentration. For instance, collagen-binding-peptide (CBD)-functionalized engineered exosomes enable high-affinity anchoring with sustained release on type-I collagen scaffolds, reducing burst release and promoting recruitment of regeneration-associated cells and axonal guidance^[142]^.

Recent studies have further explored composite systems combining exosomes with conductive and stimuli-responsive materials. Coupling exosomes with conductive hydrogels (e.g., systems containing conductive polymers) has been reported to enhance axonal growth and remyelination in rat SCI models, with immunomodulation supporting tissue repair. These systems provide mechanical support and can modulate local electrical cues, supporting multimodal neural repair^[139]^. Additionally, smart, stimuli-responsive composites - such as MXene-based photothermal hydrogels - use near-infrared photothermal conversion to provide on-demand microenvironmental modulation and tuned delivery kinetics, showing antioxidant, anti-inflammatory and pro-regenerative activities in SCI models^[165]^.

Bioinspired composite scaffolds that integrate exosomes with ECM components illustrate a shift towards local microenvironment remodeling. For example, a decellularized-ECM hydrogel combined with cortical neuron-Exos achieved localized sustained release and enrichment in rat SCI models. This system promoted tissue repair and functional recovery by biasing macrophages towards M2-like states, reducing neuronal apoptosis, activating endogenous NSCs and facilitating axonal regeneration and remyelination^[131]^. Similarly, spinal cord-derived decellularized-matrix hydrogels functionalized with exosomes showed an *in vivo* effective window of at least two weeks, reprogramming macrophages to the M2 phenotype and coordinating NSC differentiation and axonal growth^[140]^.

In summary, exosome-biomaterial composite systems provide sustained delivery, localized repair and functional regeneration via multilayer synergy. Optimizing material type, architecture and surface chemistry enables tighter control over release rates and spatial distribution and has been associated with improved neural-repair outcomes. Future work should develop intelligent composite platforms with programmable release, responsiveness to external stimuli and integrated imaging, alongside robust quality-control frameworks, to advance precision applications and clinical translation of exosome-based therapies.

## LIMITATIONS

Despite the substantial body of encouraging preclinical evidence summarized in this review, several important limitations warrant careful consideration. First, strategies designed to enhance the therapeutic efficacy of exosomes - including inflammatory or pharmacological preconditioning as well as engineering modifications - may yield divergent outcomes depending on the specific approach employed. For instance, anti-inflammatory stimulation (such as IL-4) has been shown to enhance the immunomodulatory and reparative capacity of MSC-derived exosomes, whereas proinflammatory preconditioning (such as lipopolysaccharide exposure) can generate vesicles that propagate neuroinflammation and exacerbate secondary injury. These observations underscore the necessity of context-dependent optimization of preconditioning parameters, taking into account injury stage and microenvironmental characteristics, in order to maximize therapeutic benefit while minimizing unintended adverse signaling.

Second, cross-study comparability is limited by pronounced heterogeneity in experimental design, including differences in injury models (e.g., contusion, transection, ischemia-reperfusion), injury severity, administration routes, dosing regimens, and treatment timing. These variables critically influence therapeutic outcomes and may introduce directional bias in observed effects; for example, intravenous administration is more frequently associated with systemic immunomodulation, whereas intrathecal or intraparenchymal delivery may be more favorable for localized BSCB repair. However, few studies directly compare different injury models or delivery routes within the same experimental framework, highlighting the need for standardized reporting of injury parameters, administration strategies, and functional endpoints. In addition, long-term safety data remain limited, as most studies focus on relatively short observation periods (typically 8-12 weeks). Extended follow-up will be required to systematically evaluate delayed immune responses, biodistribution profiles, and potential adverse effects relevant to clinical translation.

From a clinical and translational perspective, the feasibility of exosome-based therapies faces additional challenges. Exosomal bioactivity and safety are strongly influenced by cell source characteristics, including donor age, health status, tissue origin, and autologous *vs*. allogeneic derivation. Accumulating evidence suggests that exosomes derived from aged or metabolically compromised donors may exhibit reduced regenerative efficacy or heightened proinflammatory signaling. Moreover, current isolation and purification methods - particularly differential ultracentrifugation - are poorly suited for large-scale, Good Manufacturing Practice (GMP)-compliant production. Advances in scalable technologies, such as tangential flow filtration, size-exclusion chromatography, and microfluidic platforms, will therefore be essential to achieve reproducible clinical-grade exosome manufacturing.

Importantly, increasing efforts to engineer exosomes for enhanced targeting, cargo loading, or prolonged retention introduce additional safety considerations. While surface modification and genetic or chemical engineering strategies can improve delivery efficiency, they may also alter biodistribution patterns, promote unintended uptake by off-target tissues, or trigger unforeseen immune responses. The potential off-target effects of engineered exosomes, particularly following systemic administration, remain insufficiently characterized and warrant rigorous evaluation. In parallel, integration of exosomal biomarkers, including circulating miRNAs and transfer RNA (tRNA)-derived fragments, may facilitate patient stratification and therapeutic monitoring, thereby strengthening the translational bridge between preclinical research and clinical application.

## CONCLUSIONS

This review synthesizes current advances in EV-based therapeutic strategies for SCI, with a particular emphasis on exosome-mediated mechanisms of neural repair. By integrating evidence across diverse cellular sources, experimental models, and technological approaches, we highlight how exosomes function as active modulators of intercellular communication capable of reshaping the post-injury microenvironment, supporting axonal regeneration, and promoting functional recovery.

Collectively, the findings discussed herein underscore a broader paradigm shift in regenerative neuroscience - from cell replacement toward targeted modulation of signaling networks. As mechanistic insight deepens and engineering strategies mature, exosome-based interventions are increasingly viewed as controllable and biologically informed therapeutic platforms. With continued interdisciplinary collaboration, technological innovation, and progress toward standardized manufacturing, exosomes may emerge as important mediators of nervous system repair and hold growing promise for improving functional outcomes after SCI.

## DECLARATIONS

### AI and AI-assisted tools statement

AI and AI-assisted technologies have only been used to improve readability and language.

### Copyright

© The Author(s) 2026.
