# Supplementary material for: Extracellular vesicle-based therapeutic strategies for spinal cord injury
Source: Extracell Vesicles Circ Nucl Acids. 2026 Feb 26;7(1):259–91. doi: 10.20517/evcna.2025.142 (PMC13074289; doi:10.20517/evcna.2025.142)
Supplement: Supplementary file 1 [file evcna-7-1-259-SupplementaryMaterials.pdf]

Supplementary Materials

Extracellular vesicle-based therapeutic strategies for spinal cord injury

Jingsong Liu[1,2,3,#], Xuqiang Gong[1,2,3,#], Yuanliang Sun[1,2,3,#], Yangyang Wang[1,2,3], Yansong Wang[1,2,3]

[1]Department of Orthopedic Surgery, The First Affiliated Hospital of Harbin Medical University, Harbin Medical University, Harbin 150000, Heilongjiang, China.
[2]The Key Laboratory of Myocardial Ischemia, Ministry of Education, Harbin Medical University, Harbin 150000, Heilongjiang, China.
[3]NHC Key Laboratory of Cell Transplantation, Harbin Medical University, Harbin 150000, Heilongjiang, China.
[#]These authors contributed equally to this work.

Correspondence to: Prof. Yansong Wang, Dr. Yangyang Wang, Department of Orthopedic Surgery, The First Affiliated Hospital of Harbin Medical University, Harbin Medical University, Harbin 150000, Heilongjiang, China. E-mail: wangyansong@hrbmu.edu.cn; wangyy7790@hrbmu.edu.cn

Supplementary Table 1. Effects of Exos isolated from different MSCs in spinal cord injury

Exo Sources	Effects of Exo-administration	Animals	Ref.
BM-MSC	miR-382-5p activates the IGF-1 axis; attenuates inflammation and oxidative injury	Rat	[1]
BM-MSC	Inhibits NF-κB p65 nuclear translocation; reduces A1 astrocytes and exerts anti-inflammatory effects	Rat	[2]
BM-MSC	miR-497-5p suppresses the TXNIP/NLRP3 axis; reduces neuronal apoptosis	Rat	[3]
BM-MSC	miR-21a-5p activates the PELI1 axis to enhance autophagy; inhibits macrophage/microglial pyroptosis	Mouse	[4]
BM-MSC	Activates the Nrf2/GCH1/BH4 pathway; reduces ferroptosis	Rat	[5]
BM-MSC	miR-219-5p acts via the UBE2Z/NRF2 pathway; inhibits neuronal ferroptosis	Rat	[6]
BM-MSC	Inhibits the IL-17 pathway; suppresses ferroptosis	Rat	[7]
BM-MSC	Delivers ZBTB4 to inhibit ITIH3; alleviates astrocyte-induced neuronal injury	Mouse	[8]
BM-MSC	Up-regulates TGF-β in M2 macrophages; decreases BSCB permeability	Rat	[9]
BM-MSC	Reduces neuronal death and down-regulates NF-κB p65 signaling; inhibits pericyte migration and lowers BSCB permeability	Rat	[10]
BM-MSC	Modulates the NOD1-related pathway to promote pericyte survival and inhibit pyroptosis; maintains BSCB integrity	Rat	[11]
BM-MSC	Promotes endothelial proliferation, migration, and angiogenesis; reduces neuronal apoptosis; attenuates inflammation; suppresses A1 astrocyte activation	Rat	[12]
BM-MSC	Delivers let-7a-5p targeting the HMGA2/SMAD2 axis; promotes neural stem cell differentiation into neurons	Rat	[13]
BM-MSC	miR-431-3p/RGMA axis facilitates axonal regeneration	Mouse	[14]
UC-MSC	Inhibits NF-κB/MAPK signaling; reduces inflammation	Rat	[15]
UC-MSC	miR-146b acts via the TLR4-mediated NF-κB p65 pathway; alleviates inflammation	Rat	[16]

UC-MSC	Modulates the BCL-2/Bax balance and activates the Wnt/β-catenin pathway; exerts anti-apoptotic and anti-inflammatory effects	Rat	[17]
UC-MSC	Mitigates junctional disruption by down-regulating MMP-2/MMP-9 and inhibiting ET-1; promotes BSCB repair	Rat	[18]
UC-MSC	Promotes neurite outgrowth; reduces inflammation	Rat	[19]
UC-MSC	Reduces apoptosis and inflammation; prevents axonal loss; enhances angiogenesis; decreases fibrosis	Mouse	[20]
PMSC	Attenuates inflammation and oxidative injury	Rat	[21]
PMSC	Activates the MEK/ERK/CREB pathway to stimulate NSC proliferation; promotes neurogenesis by activating endogenous progenitors	Rat	[22]
ADSC	Attenuates inflammation	Rat	[23]

BM-MSC: Bone marrow mesenchymal stem cell; UC-MSC: umbilical cord mesenchymal stem cell; PMSC: placenta-derived mesenchymal stem cell; ADSC: adipose-derived mesenchymal stem cell; OM-MSC: olfactory mucosa-derived mesenchymal stem cell.

Supplementary Table 2. Effects of Exos isolated from different immune cells in spinal cord injury

Exo Sources	Effects of Exo-administration	Animals	Ref.
Macrophage	miR-151-3p targets P53 to modulate the p53/p21/CDK1 axis and reduce neuronal apoptosis	Mouse	[24]
M1-Macrophage	miR-155 activates NF-κB signaling, inducing EndoMT and mitochondrial dysfunction in endothelial cells after SCI	Mouse	[25]
M2-Macrophage	miR-2861 promotes blood-spinal cord barrier repair and motor function restoration	Mouse	[26]
Regulatory T cells	Suppresses A1 astrocyte activation via NF-κB/p65 inhibition	Mouse	[27]
M2-Microglial	miR-421-3p-enriched exosomes inhibit mTOR signaling to attenuate neuronal apoptosis	Mouse	[28]
M2-Microglial	miR-709/NKAP axis reduces microglial pyroptosis after SCI	Mouse	[29]

Supplementary Table 3. Effects of Exos isolated from different neural cells in spinal cord injury

Exo Sources	Effects of Exo-administration	Animals	Ref.
NSC	miR-374-5p targets SKT-4 to balance apoptosis and autophagy	Mouse	[30]
NSC	miR-34a-5p downregulates HDAC6 to stabilize microtubules and induce autophagy	Mouse	[31]
NSC	ASIC1A/PTGS2/PGE2 axis inhibits NSC-oligodendrocyte differentiation	Rat	[32]
NSC	VEGF-A exosomes enhance endothelial migration and angiogenesis	Mouse	[33]
Neuron	miR-124-3p/MYH9 axis modulates post-SCI immune response	Mouse	[34]
Neuron	Suppresses glial activation; promotes axon growth, NSC differentiation, and remyelination	Mouse	[35]
Neuron	Inhibits M1 macrophages; Rab3a/STXBP1-METTL2b regulate EV secretion and inflammation	Rat	[36]
Astrocyte	Rab27a-Rho/ROCK signaling promotes CSPG deposition and glial scar	Rat	[37]
Astrocyte	Hippo pathway activation promotes neurite outgrowth and motor recovery	Rat	[38]
Astrocyte	Astrocyte-sEVs (CCL2/CCR2) activate microglia and IL-1β release	Rat	[39]
SC	TLR2 activates NF-κB/PI3K signaling to modulate astrocytic immunity	Mouse	[40]
SC	EGFR/Akt/mTOR activation enhances autophagy and reduces apoptosis	Rat	[41]
SC	MFG-E8/SOCS3/STAT3 signaling regulates macrophage-microglia polarization	Rat	[42]
SC	AMPK activation preserves mitochondria and limits oxidative stress	Rat	[43]
SC	Rho/ROCK-PTP-σ modulation reduces scarring and improves recovery	Mouse	[44]
OEC	Promotes neural progenitor proliferation; reduces oxidative toxicity	Neural precursor cells(NPC)	[45]

NSC: Neural stem cell; SC: schwann cell; OEC: olfactory ensheathing cell.

Supplementary Table 4. Effects of Exos isolated from other Sources

Exo Sources	Effects of Exo-administration	Animals	Ref.
Endothelial	Promoted neural stem cell differentiation into neurons via L1CAM-Akt activation, enhanced neuroregeneration	Mouse	[46]
EPCs	Enhanced M2 macrophage polarization via miR-222-3p/SOCS3/JAK2/STAT3 activation	Mouse	[47]
iPSC	Induced M2 macrophage polarization, decreased pro-inflammatory and increased anti-inflammatory factors via miR-199b-5p/Hgf-PI3K pathway	Mouse	[48]
iPSC	Inhibited microglial and macrophage pyroptosis, preserved myelin integrity, promoted axonal growth via let-7b-5p/LRIG3 axis	Mouse	[49]
iPSC	Enhanced early M2 polarization, reduced neuronal apoptosis, promoted axonal regeneration and remyelination, improved motor and urinary function	Rat	[50]
Plant	Reduced inflammation, promoted axonal repair and neuronal differentiation; ISL-loaded vesicles achieved targeted drug delivery	Rat	[51]
Plasma	Elevated levels of CD47, CD56, CD68, and ADAM17 in body fluids	Human	[52]
Plasma	Downregulation of miR-429 increased neuronal apoptosis; reduced PTEN/PI3K/Akt pathway activity	Rat	[53]
Plasma	Increased $CD81^{+}$ exosomes in plasma and decreased CD81 expression in astrocytes	Mouse	[54]
Plasma	Aged SCI exosomes showed upregulated pro-inflammatory factors and neuronal apoptosis, young SCI exosomes exhibited increased protective molecules	Mouse	[55]
Plasma	Upregulation of CXCL2, IL-6, and caspase-3 in exosomes, with downregulated autophagy-related miRNAs and impaired autophagy	Mouse	[56]
Plasma	Differentially expressed lncRNAs in exosomes formed lncRNA-miRNA-mRNA regulatory	Rat	[57]

	networks related to inflammation, apoptosis, and regeneration		
Plasma	In female SCI mice, increased microglial and myeloid cell infiltration and elevated ROS/TNF expression;<br>In males, plasma exosomal markers were upregulated	Mouse	[58]
Plasma	Increased CD9 expression and abnormal expression of Grm1, Nrg1, CD63, Enpp3, and Cxcr4 during the acute phase	Rat	[59]
Plasma	Enhanced RVG-mediated neuronal targeting, increased ILP/ISP signaling, and improved axonal regeneration and motor function	Mouse	[60]
Plasma	Improved axonal myelination, reduced lesion cavity area, and enhanced functional recovery	Rat	[61]
Plasma	Altered circRNA-miRNA-mRNA network expression regulating inflammation and tissue repair	Rat	[62]
PRP	Enhanced neuroprotective signaling and decreased pro-inflammatory cytokine expression	Rat	[63]
PRP	Upregulated tight junction proteins, decreased blood-spinal cord barrier permeability, reduced NF-κB activity, and attenuated inflammation	Rat	[64]
Spinal cord tissue	LPS-induced exosomes activated astrocytes and participated in inflammatory signaling loops	Mouse	[65]

Endothelial: Endothelial cell; EPC: endothelial progenitor cell; iPSC: induced pluripotent stem cell; Plant: plant cell; PRP: platelet-rich plasma; Serum: serum; SVZ: subventricular zone.

Supplementary Table 5. Enhancing the therapeutic efficacy of exosomes for spinal cord injury through cell preconditioning

Exo Sources	Preconditioning method	Effects of Exo-administration	Animals	Ref.
UC-MSC	Hypoxia	Enhanced tube formation and anti-inflammatory response, promoted neuroregeneration	Mouse	[66]
ADSC-Exos	Hypoxia	Reduced neuronal apoptosis and cavity formation; miR-499a-5p inhibited JNK3/c-Jun pathway	Mouse	[67]
BMSCs	Hypoxia	M1→M2 macrophage shift; miR-146a-5p targeted IRAK1 to regulate polarization	Rat	[68]
BMSCs	Hypoxia	M1→M2 microglial polarization; miR-216a-5p regulated TLR4/NF-κB/PI3K/AKT pathway	Mouse	[69]
MSC	Hypoxia	Upregulated HIF-1α/VEGF, enhanced angiogenesis	Rat	[70]
NSC	Hypoxia	HIF-1α/RAB17 activation promoted EV secretion and repair; CAQK/Angiopep2 improved spinal targeting	Rat	[71]
MSC	Hypoxia	Increased SOD/T-AOC, decreased MDA; activated SIRT1/Nrf2/HO-1; upregulated Bcl-2, reduced apoptosis	Rat	[72]
ADSC-Exos	Hypoxia	circ-Astn1 activated miR-138-5p/Atg7 axis, enhanced autophagy, reduced inflammation	Rat	[73]
ADSC-Exos	Hypoxia	lncGm37494 suppressed miR-130b-3p, upregulated PPARγ, induced M2 polarization	Rat	[74]
ADSC-Exos	Hypoxia	circ-Wdfy3 targeted miR-423-3p/GPX4 axis, inhibited ferroptosis, decreased ROS	Rat	[75]
UC-MSC	Hypoxia	Downregulated IL-1β/IL-6/TNF-α; activated circOXNAD1/miR-29a-3p/FOXO3a axis	Rat	[76]

BMSCs	Hypoxia	A1 astrocytes ↓, A2 ↑, neuronal apoptosis ↓; miR-21/JAK2/STAT3 modulated	Rat	[77]
Microglia	LPS	Regulated microglia–astrocyte interaction; Smad3 ↓; scar formation inhibited via miR-145-5p/Smad3 axis	Rat	[78]
Microglia	Melatonin	Anti-inflammatory effect ↑; phagocytosis and BSCB integrity improved; promoted remyelination	Mouse	[79]
BMSCs	Melatonin	Melatonin induced, M1→M2 shift, reduced ROS, stabilized NRF2 via USP29; improved motor recovery	Mouse	[80]

↑ Indicates upregulation; ↓ indicates downregulation; → indicates a change in the direction of the phenotypic outcome. UC-MSC: Umbilical cord mesenchymal stem cell; ADSC: adipose-derived mesenchymal stem cell; BMSC: bone marrow mesenchymal stem cell; MSC: mesenchymal stem cell; NSC: neural stem cell.

REFERENCES

1. Yang D, Wei H, Sheng Y, Peng T, Zhao Q, et al. Circ_0006640 transferred by bone marrow-mesenchymal stem cell-exosomes suppresses lipopolysaccharide-induced apoptotic, inflammatory and oxidative injury in spinal cord injury. *J Orthop Surg Res* 2024;19:50. DOI:10.1186/s13018-023-04523-9
2. Wang L, Pei S, Han L, Guo B, Li Y, et al. Mesenchymal Stem Cell-Derived Exosomes Reduce A1 Astrocytes via Downregulation of Phosphorylated NFκB P65 Subunit in Spinal Cord Injury. *Cell Physiol Biochem* 2018;50:1535-59. DOI:10.1159/000494652
3. Xu J, Zhang J, Liu Q, Wang B. Bone marrow mesenchymal stem cells-derived exosomes promote spinal cord injury repair through the miR-497-5p/TXNIP/NLRP3 axis. *J Mol Histol* 2024;56:16. DOI:10.1007/s10735-024-10289-z
4. Gu J, Wu J, Wang C, Xu Z, Jin Z, et al. BMSCs-derived exosomes inhibit macrophage/microglia pyroptosis by increasing autophagy through the miR-21a-5p/PELI1 axis in spinal cord injury. *Aging (Albany NY)* 2024;16:5184-206. DOI:10.18632/aging.205638
5. Chen Y, Li B, Quan J, Li Z, Li Y, et al. Inhibition of Ferroptosis by Mesenchymal Stem Cell-Derived Exosomes in Acute Spinal Cord Injury: Role of Nrf2/GCH1/BH4 Axis. *Neurospine* 2024;21:642-55. DOI:10.14245/ns.2448038.019
6. Dong J, Gong Z, Bi H, Yang J, Wang B, et al. BMSC-derived exosomal miR-219-5p alleviates ferroptosis in neuronal cells caused by spinal cord injury via the UBE2Z/NRF2 pathway. *Neuroscience* 2024;556:73-85. DOI:10.1016/j.neuroscience.2024.06.011
7. Tang W, Zhao K, Li X, Zhou X, Liao P. Bone Marrow Mesenchymal Stem Cell-Derived Exosomes Promote the Recovery of Spinal Cord Injury and Inhibit Ferroptosis by Inactivating IL-17 Pathway. *J Mol Neurosci* 2024;74:33. DOI:10.1007/s12031-024-02209-3
8. Wu H, Wang Q, Liao Y, Wang S. MSC-derived exosomes deliver ZBTB4 to mediate transcriptional repression of ITIH3 in astrocytes in spinal cord injury. *Brain Res Bull* 2024;212:110954. DOI:10.1016/j.brainresbull.2024.110954
9. Nakazaki M, Morita T, Lankford KL, Askenase PW, Kocsis JD. Small extracellular vesicles released by infused mesenchymal stromal cells target M2 macrophages and promote TGF-β upregulation, microvascular stabilization and functional recovery in a rodent model of severe spinal cord injury. *J Extracell Vesicles* 2021;10:e12137. DOI:10.1002/jev2.12137
10. Lu Y, Zhou Y, Zhang R, Wen L, Wu K, et al. Bone Mesenchymal Stem Cell-Derived Extracellular Vesicles Promote Recovery Following Spinal Cord Injury via Improvement of the Integrity of the Blood-Spinal Cord Barrier. *Front Neurosci* 2019;13:209. DOI:10.3389/fnins.2019.00209
11. Zhou Y, Wen LL, Li YF, Wu KM, Duan RR, et al. Exosomes derived from bone marrow mesenchymal stem cells protect the injured spinal cord by inhibiting pericyte pyroptosis. *Neural Regen Res* 2022;17:194-202. DOI:10.4103/1673-5374.314323
12. Liu W, Wang Y, Gong F, Rong Y, Luo Y, et al. Exosomes Derived from Bone Mesenchymal Stem Cells Repair Traumatic Spinal Cord Injury by Suppressing the

Activation of A1 Neurotoxic Reactive Astrocytes. *J Neurotrauma* 2019;36:469-84. DOI:10.1089/neu.2018.5835
13. Wang Y, Han T, Guo R, Song P, Liu Y, et al. Micro-RNA let-7a-5p Derived From Mesenchymal Stem Cell-Derived Extracellular Vesicles Promotes the Regrowth of Neurons in Spinal-Cord-Injured Rats by Targeting the HMGA2/SMAD2 Axis. *Front Mol Neurosci* 2022;15:850364. DOI:10.3389/fnmol.2022.850364
14. Sun Y, Liu Q, Qin Y, Xu Y, Zhao J, et al. Exosomes derived from CD271(+)CD56(+) bone marrow mesenchymal stem cell subpopoulation identified by single-cell RNA sequencing promote axon regeneration after spinal cord injury. *Theranostics* 2024;14:510-27. DOI:10.7150/thno.89008
15. Luan Z, Liu J, Li M, Wang Y, Wang Y. Exosomes derived from umbilical cord-mesenchymal stem cells inhibit the NF-κB/MAPK signaling pathway and reduce the inflammatory response to promote recovery from spinal cord injury. *J Orthop Surg Res* 2024;19:184. DOI:10.1186/s13018-024-04651-w
16. Wang X, Yang Y, Li W, Hao M, Xu Y. Umbilical mesenchymal stem cell-derived exosomes promote spinal cord functional recovery through the miR-146b/TLR4 -mediated NF-κB p65 signaling pathway in rats. *Biochem Biophys Rep* 2023;35:101497. DOI:10.1016/j.bbrep.2023.101497
17. Kang J, Guo Y. Human Umbilical Cord Mesenchymal Stem Cells Derived Exosomes Promote Neurological Function Recovery in a Rat Spinal Cord Injury Model. *Neurochem Res* 2022;47:1532-40. DOI:10.1007/s11064-022-03545-9
18. Xue C, Ma X, Guan X, Feng H, Zheng M, et al. Small extracellular vesicles derived from umbilical cord mesenchymal stem cells repair blood-spinal cord barrier disruption after spinal cord injury through down-regulation of Endothelin-1 in rats. *PeerJ* 2023;11:e16311. DOI:10.7717/peerj.16311
19. Wang Y, Lai X, Wu D, Liu B, Wang N, et al. Umbilical mesenchymal stem cell-derived exosomes facilitate spinal cord functional recovery through the miR-199a-3p/145-5p-mediated NGF/TrkA signaling pathway in rats. *Stem Cell Res Ther* 2021;12:117. DOI:10.1186/s13287-021-02148-5
20. Lee JR, Kyung JW, Kumar H, Kwon SP, Song SY, et al. Targeted Delivery of Mesenchymal Stem Cell-Derived Nanovesicles for Spinal Cord Injury Treatment. *Int J Mol Sci* 2020;21. DOI:10.3390/ijms21114185
21. Li L, Zhang Y, Mu J, Chen J, Zhang C, et al. Transplantation of Human Mesenchymal Stem-Cell-Derived Exosomes Immobilized in an Adhesive Hydrogel for Effective Treatment of Spinal Cord Injury. *Nano Lett* 2020;20:4298-305. DOI:10.1021/acs.nanolett.0c00929
22. Zhou W, Silva M, Feng C, Zhao S, Liu L, et al. Exosomes derived from human placental mesenchymal stem cells enhanced the recovery of spinal cord injury by activating endogenous neurogenesis. *Stem Cell Res Ther* 2021;12:174. DOI:10.1186/s13287-021-02248-2
23. Sung SE, Seo MS, Kim YI, Kang KK, Choi JH, et al. Human Epidural AD-MSC Exosomes Improve Function Recovery after Spinal Cord Injury in Rats. *Biomedicines* 2022;10. DOI:10.3390/biomedicines10030678
24. Li C, Qin T, Liu Y, Wen H, Zhao J, et al. Microglia-Derived Exosomal microRNA-

151-3p Enhances Functional Healing After Spinal Cord Injury by Attenuating Neuronal Apoptosis via Regulating the p53/p21/CDK1 Signaling Pathway. *Front Cell Dev Biol* 2021;9:783017. DOI:10.3389/fcell.2021.783017
25. Ge X, Tang P, Rong Y, Jiang D, Lu X, et al. Exosomal miR-155 from M1-polarized macrophages promotes EndoMT and impairs mitochondrial function via activating NF-κB signaling pathway in vascular endothelial cells after traumatic spinal cord injury. *Redox Biol* 2021;41:101932. DOI:10.1016/j.redox.2021.101932
26. Kong G, Xiong W, Li C, Xiao C, Wang S, et al. Treg cells-derived exosomes promote blood-spinal cord barrier repair and motor function recovery after spinal cord injury by delivering miR-2861. *J Nanobiotechnology* 2023;21:364. DOI:10.1186/s12951-023-02089-6
27. Zhang J, Hu D, Li L, Qu D, Shi W, et al. M2 Microglia-derived Exosomes Promote Spinal Cord Injury Recovery in Mice by Alleviating A1 Astrocyte Activation. *Mol Neurobiol* 2024;61:7009-25. DOI:10.1007/s12035-024-04026-6
28. Wang J, Rong Y, Ji C, Lv C, Jiang D, et al. MicroRNA-421-3p-abundant small extracellular vesicles derived from M2 bone marrow-derived macrophages attenuate apoptosis and promote motor function recovery via inhibition of mTOR in spinal cord injury. *J Nanobiotechnology* 2020;18:72. DOI:10.1186/s12951-020-00630-5
29. Xiong W, Li C, Kong G, Zeng Q, Wang S, et al. Treg cell-derived exosomes miR-709 attenuates microglia pyroptosis and promotes motor function recovery after spinal cord injury. *J Nanobiotechnology* 2022;20:529. DOI:10.1186/s12951-022-01724-y
30. Zhang L, Han P. Neural stem cell-derived exosomes suppress neuronal cell apoptosis by activating autophagy via miR-374-5p/STK-4 axis in spinal cord injury. *J Musculoskelet Neuronal Interact* 2022;22:411-21
31. Qin T, Li C, Xu Y, Qin Y, Jin Y, et al. Local delivery of EGFR(+)NSCs-derived exosomes promotes neural regeneration post spinal cord injury via miR-34a-5p/HDAC6 pathway. *Bioact Mater* 2024;33:424-43. DOI:10.1016/j.bioactmat.2023.11.013
32. Wu Z, Han T, Dong Y, Ying W, Fang H, et al. Acid-sensing ion channel-1 contributes to the failure of myelin sheath regeneration following spinal cord injury by transcellular delivery of PGE2. *Cell Mol Biol Lett* 2024;29:149. DOI:10.1186/s11658-024-00672-9
33. Zhong D, Cao Y, Li CJ, Li M, Rong ZJ, et al. Neural stem cell-derived exosomes facilitate spinal cord functional recovery after injury by promoting angiogenesis. *Exp Biol Med (Maywood)* 2020;245:54-65. DOI:10.1177/1535370219895491
34. Jiang D, Gong F, Ge X, Lv C, Huang C, et al. Neuron-derived exosomes-transmitted miR-124-3p protect traumatically injured spinal cord by suppressing the activation of neurotoxic microglia and astrocytes. *J Nanobiotechnology* 2020;18:105. DOI:10.1186/s12951-020-00665-8
35. Xu Y, Zhu ZH, Xu X, Sun HT, Zheng HM, et al. Neuron-Derived Exosomes Promote the Recovery of Spinal Cord Injury by Modulating Nerve Cells in the Cellular Microenvironment of the Lesion Area. *Mol Neurobiol* 2023;60:4502-16. DOI:10.1007/s12035-023-03341-8
36. Yang Y, Liu Z, Lu Y, Yu X, Zhu R, et al. Rab3a attenuates spinal cord injury by

mediating vesicle release. *Brain Res Bull* 2024;208:110884. DOI:10.1016/j.brainresbull.2024.110884
37. Singh N, Pathak Z, Kumar H. Rab27a-mediated extracellular vesicle release drives astrocytic CSPG secretion and glial scarring in spinal cord injury. *Biomater Adv* 2025;176:214357. DOI:10.1016/j.bioadv.2025.214357
38. Sun H, Cao X, Gong A, Huang Y, Xu Y, et al. Extracellular vesicles derived from astrocytes facilitated neurite elongation by activating the Hippo pathway. *Exp Cell Res* 2022;411:112937. DOI:10.1016/j.yexcr.2021.112937
39. Rong Y, Ji C, Wang Z, Ge X, Wang J, et al. Small extracellular vesicles encapsulating CCL2 from activated astrocytes induce microglial activation and neuronal apoptosis after traumatic spinal cord injury. *J Neuroinflammation* 2021;18:196. DOI:10.1186/s12974-021-02268-y
40. Pan D, Li Y, Yang F, Lv Z, Zhu S, et al. Increasing toll-like receptor 2 on astrocytes induced by Schwann cell-derived exosomes promotes recovery by inhibiting CSPGs deposition after spinal cord injury. *J Neuroinflammation* 2021;18:172. DOI:10.1186/s12974-021-02215-x
41. Pan D, Zhu S, Zhang W, Wei Z, Yang F, et al. Autophagy induced by Schwann cell-derived exosomes promotes recovery after spinal cord injury in rats. *Biotechnol Lett* 2022;44:129-42. DOI:10.1007/s10529-021-03198-8
42. Ren J, Zhu B, Gu G, Zhang W, Li J, et al. Schwann cell-derived exosomes containing MFG-E8 modify macrophage/microglial polarization for attenuating inflammation via the SOCS3/STAT3 pathway after spinal cord injury. *Cell Death Dis* 2023;14:70. DOI:10.1038/s41419-023-05607-4
43. Xu B, Zhou Z, Fang J, Wang J, Tao K, et al. Exosomes derived from schwann cells alleviate mitochondrial dysfunction and necroptosis after spinal cord injury via AMPK signaling pathway-mediated mitophagy. *Free Radic Biol Med* 2023;208:319-33. DOI:10.1016/j.freeradbiomed.2023.08.026
44. Zhu S, Ma H, Hou M, Li H, Ning G. Schwann Cell-Derived Exosomes Induced Axon Growth after Spinal Cord Injury by Decreasing PTP-σ Activation on CSPGs via the Rho/ROCK Pathway. *Neurochem Res* 2024;49:2120-30. DOI:10.1007/s11064-024-04166-0
45. Tu YK, Hsueh YH. Extracellular vesicles isolated from human olfactory ensheathing cells enhance the viability of neural progenitor cells. *Neurol Res* 2020;42:959-67. DOI:10.1080/01616412.2020.1794371
46. Liu Y, Luo Z, Xie Y, Sun Y, Yuan F, et al. Extracellular vesicles from UTX-knockout endothelial cells boost neural stem cell differentiation in spinal cord injury. *Cell Commun Signal* 2024;22:155. DOI:10.1186/s12964-023-01434-4
47. Yuan F, Peng W, Yang Y, Xu J, Liu Y, et al. Endothelial progenitor cell-derived exosomes promote anti-inflammatory macrophages via SOCS3/JAK2/STAT3 axis and improve the outcome of spinal cord injury. *J Neuroinflammation* 2023;20:156. DOI:10.1186/s12974-023-02833-7
48. Li J, Jing Y, Bai F, Wu Y, Wang L, et al. Induced pluripotent stem cells as natural biofactories for exosomes carrying miR-199b-5p in the treatment of spinal cord injury. *Front Pharmacol* 2022;13:1078761. DOI:10.3389/fphar.2022.1078761

49. Liu J, Kong G, Lu C, Wang J, Li W, et al. IPSC-NSCs-derived exosomal let-7b-5p improves motor function after spinal cord Injury by modulating microglial/macrophage pyroptosis. *J Nanobiotechnology* 2024;22:403. DOI:10.1186/s12951-024-02697-w
50. Wang G, Li Q, Liu S, Li M, Liu B, et al. An injectable decellularized extracellular matrix hydrogel with cortical neuron-derived exosomes enhances tissue repair following traumatic spinal cord injury. *Mater Today Bio* 2024;28:101250. DOI:10.1016/j.mtbio.2024.101250
51. Wang Q, Liu K, Cao X, Rong W, Shi W, et al. Plant-derived exosomes extracted from Lycium barbarum L. loaded with isoliquiritigenin to promote spinal cord injury repair based on 3D printed bionic scaffold. *Bioeng Transl Med* 2024;9:e10646. DOI:10.1002/btm2.10646
52. Hörauf JA, Schindler CR, Schaible I, Wang M, Weber B, et al. Extracellular vesicles epitopes as potential biomarker candidates in patients with traumatic spinal cord injury. *Front Immunol* 2024;15:1478786. DOI:10.3389/fimmu.2024.1478786
53. Huang J, Wu C, Xu G, Sun Y, Gui C, et al. The decreased expression of miR-429 in plasma exosomes after spinal cord injury inhibits neuronal apoptosis by mediating the PTEN/PI3K/Akt pathway. *Ann Transl Med* 2022;10:6. DOI:10.21037/atm-21-5561
54. Khan NZ, Cao T, He J, Ritzel RM, Li Y, et al. Spinal cord injury alters microRNA and CD81+ exosome levels in plasma extracellular nanoparticles with neuroinflammatory potential. *Brain Behav Immun* 2021;92:165-83. DOI:10.1016/j.bbi.2020.12.007
55. Lei Z, Krishnamachary B, Khan NZ, Ji Y, Li Y, et al. Spinal cord injury disrupts plasma extracellular vesicles cargoes leading to neuroinflammation in the brain and neurological dysfunction in aged male mice. *Brain Behav Immun* 2024;120:584-603. DOI:10.1016/j.bbi.2024.07.005
56. Lei Z, Krishnamachary B, Ritzel RM, Khan NZ, Li Y, et al. Age-related changes in plasma extracellular vesicles influence neuroinflammation in the brain and neurological outcome after traumatic spinal cord injury. *Res Sq* 2023 DOI:10.21203/rs.3.rs-2821858/v1
57. Li JA, Shi MP, Cong L, Gu MY, Chen YH, et al. Circulating exosomal lncRNA contributes to the pathogenesis of spinal cord injury in rats. *Neural Regen Res* 2023;18:889-94. DOI:10.4103/1673-5374.353504
58. Li Y, Khan N, Ritzel RM, Lei Z, Allen S, et al. Sexually dimorphic extracellular vesicle responses after chronic spinal cord injury are associated with neuroinflammation and neurodegeneration in the aged brain. *J Neuroinflammation* 2023;20:197. DOI:10.1186/s12974-023-02881-z
59. Mirzaalikhan Y, Eslami N, Izadi A, Shekari F, Kiani S. Spinal Cord Injury Affects Gene Expression of Transmembrane Proteins in Tissue and Release of Extracellular Vesicle in Blood: In Silico and In Vivo Analysis. *Cell J* 2023;25:772-82. DOI:10.22074/cellj.2023.2004115.1320
60. Ran N, Li W, Zhang R, Lin C, Zhang J, et al. Autologous exosome facilitates load and target delivery of bioactive peptides to repair spinal cord injury. *Bioact Mater* 2023;25:766-82. DOI:10.1016/j.bioactmat.2022.07.002
61. Zamanian C, Onyedimma C, Moinuddin FM, Ghaith AK, Jarrah R, et al.

Evaluating purified exosome product and its role in neurologic and functional recovery following spinal cord injury in female rats. *J Spinal Cord Med* 2025;48:527-35. DOI:10.1080/10790268.2023.2274637
62. Zan C, Li J, Lin F, Wang Z. Potential value of differentially expressed circular RNAs derived from circulating exosomes in the pathogenesis of rat spinal cord injury. *Front Neurosci* 2022;16:1003628. DOI:10.3389/fnins.2022.1003628
63. Akbari-Gharalari N, Ghahremani-Nasab M, Naderi R, Aliyari-Serej Z, Karimipour M, et al. Improvement of spinal cord injury symptoms by targeting the Bax/Bcl2 pathway and modulating TNF-α/IL-10 using Platelet-Rich Plasma exosomes loaded with dexamethasone. *AIMS Neurosci* 2023;10:332-53. DOI:10.3934/Neuroscience.2023026
64. Nie X, Liu Y, Yuan T, Yu T, Yun Z, et al. Platelet-rich plasma-derived exosomes promote blood-spinal cord barrier repair and attenuate neuroinflammation after spinal cord injury. *J Nanobiotechnology* 2024;22:456. DOI:10.1186/s12951-024-02737-5
65. Memo C, Parisse P, Amoriello R, Pachetti M, Palandri A, et al. Extracellular vesicles released by LPS-stimulated spinal organotypic slices spread neuroinflammation into naïve slices through connexin43 hemichannel opening and astrocyte aberrant calcium dynamics. *Front Cell Neurosci* 2024;18:1433309. DOI:10.3389/fncel.2024.1433309
66. Li L, Mu J, Zhang Y, Zhang C, Ma T, et al. Stimulation by Exosomes from Hypoxia Preconditioned Human Umbilical Vein Endothelial Cells Facilitates Mesenchymal Stem Cells Angiogenic Function for Spinal Cord Repair. *ACS Nano* 2022;16:10811-23. DOI:10.1021/acsnano.2c02898
67. Liang Y, Wu JH, Zhu JH, Yang H. Exosomes Secreted by Hypoxia-Pre-conditioned Adipose-Derived Mesenchymal Stem Cells Reduce Neuronal Apoptosis in Rats with Spinal Cord Injury. *J Neurotrauma* 2022;39:701-14. DOI:10.1089/neu.2021.0290
68. Liang Z, Yang Z, Xie H, Rao J, Xu X, et al. Small extracellular vesicles from hypoxia-preconditioned bone marrow mesenchymal stem cells attenuate spinal cord injury via miR-146a-5p-mediated regulation of macrophage polarization. *Neural Regen Res* 2024;19:2259-69. DOI:10.4103/1673-5374.391194
69. Liu W, Rong Y, Wang J, Zhou Z, Ge X, et al. Exosome-shuttled miR-216a-5p from hypoxic preconditioned mesenchymal stem cells repair traumatic spinal cord injury by shifting microglial M1/M2 polarization. *J Neuroinflammation* 2020;17:47. DOI:10.1186/s12974-020-1726-7
70. Mu J, Li L, Wu J, Huang T, Zhang Y, et al. Hypoxia-stimulated mesenchymal stem cell-derived exosomes loaded by adhesive hydrogel for effective angiogenic treatment of spinal cord injury. *Biomater Sci* 2022;10:1803-11. DOI:10.1039/d1bm01722e
71. Qin T, Qin Y, Wen H, Wu T, Duan C, et al. Hypoxic Neural Stem Cells Enhance Spinal Cord Repair Through HIF-1a/RAB17-Driven Extracellular Vesicle Release. *J Extracell Vesicles* 2025;14:e70126. DOI:10.1002/jev2.70126
72. Rao J, Xie H, Liang Z, Yang Z, Chen P, et al. Hypoxic-preconditioned mesenchymal stem cell-derived small extracellular vesicles inhibit neuronal death after spinal cord injury by regulating the SIRT1/Nrf2/HO-1 pathway. *Front Pharmacol* 2024;15:1419390. DOI:10.3389/fphar.2024.1419390

73. Shao M, Jin M, Feizhou L, Ma X, Wei Z. Administration of hypoxic pretreated adipose-derived mesenchymal stem cell exosomes promotes spinal cord repair after injury via delivery of circ-Astn1 and activation of autophagy. *Int Immunopharmacol* 2025;152:114324. DOI:10.1016/j.intimp.2025.114324
74. Shao M, Jin M, Xu S, Zheng C, Zhu W, et al. Exosomes from Long Noncoding RNA-Gm37494-ADSCs Repair Spinal Cord Injury via Shifting Microglial M1/M2 Polarization. *Inflammation* 2020;43:1536-47. DOI:10.1007/s10753-020-01230-z
75. Shao M, Ye S, Chen Y, Yu C, Zhu W. Exosomes from hypoxic ADSCs ameliorate neuronal damage post spinal cord injury through circ-Wdfy3 delivery and inhibition of ferroptosis. *Neurochem Int* 2024;177:105759. DOI:10.1016/j.neuint.2024.105759
76. Wang X, Li W, Hao M, Yang Y, Xu Y. Hypoxia-treated umbilical mesenchymal stem cell alleviates spinal cord ischemia-reperfusion injury in SCI by circular RNA circOXNAD1/ miR-29a-3p/ FOXO3a axis. *Biochem Biophys Rep* 2023;34:101458. DOI:10.1016/j.bbrep.2023.101458
77. Yang Z, Liang Z, Rao J, Xie H, Zhou M, et al. Hypoxic-preconditioned mesenchymal stem cell-derived small extracellular vesicles promote the recovery of spinal cord injury by affecting the phenotype of astrocytes through the miR-21/JAK2/STAT3 pathway. *CNS Neurosci Ther* 2024;30:e14428. DOI:10.1111/cns.14428
78. Ye Y, Hao J, Hong Z, Wu T, Ge X, et al. Downregulation of MicroRNA-145-5p in Activated Microglial Exosomes Promotes Astrocyte Proliferation by Removal of Smad3 Inhibition. *Neurochem Res* 2022;47:382-93. DOI:10.1007/s11064-021-03446-3
79. Kong G, Liu J, Wang J, Yu X, Li C, et al. Engineered Extracellular Vesicles Modified by Angiopep-2 Peptide Promote Targeted Repair of Spinal Cord Injury and Brain Inflammation. *ACS Nano* 2025;19:4582-600. DOI:10.1021/acsnano.4c14675
80. Liu W, Tang P, Wang J, Ye W, Ge X, et al. Extracellular vesicles derived from melatonin-preconditioned mesenchymal stem cells containing USP29 repair traumatic spinal cord injury by stabilizing NRF2. *J Pineal Res* 2021;71:e12769. DOI:10.1111/jpi.12769
